# Impairment of hippocampal gamma oscillations, mitochondria and neurovascular function in CADASIL

**DOI:** 10.1093/brain/awag033

**Published:** 2026-02-02

**Authors:** Wenchao Shao, Daniel V Oliveira, Luana Naia, Yue Li, Katrine D Bjørnholm, Arturo G Isla, Per Uhlén, Raj Kalaria, Saskia A J Lesnik Oberstein, Urban Lendahl, Luis Enrique Arroyo-García, ShaoBo Jin, Helena Karlström

**Affiliations:** Department of Neurobiology, Care Science and Society, Division of Neurogeriatrics, Karolinska Institutet, Solna SE-171 64, Sweden; Department of Neurobiology, Care Science and Society, Division of Neurogeriatrics, Karolinska Institutet, Solna SE-171 64, Sweden; Faculty of Science, Department of Cell Biology, Charles University, Prague 12 000, Czech Republic; Department of Neurobiology, Care Science and Society, Division of Neurogeriatrics, Karolinska Institutet, Solna SE-171 64, Sweden; Department of Medical Biochemistry and Biophysics, Karolinska Institutet, Solna SE-171 64, Sweden; Department of Neurobiology, Care Science and Society, Division of Neurogeriatrics, Karolinska Institutet, Solna SE-171 64, Sweden; Department of Neurobiology, Care Science and Society, Division of Neurogeriatrics, Karolinska Institutet, Solna SE-171 64, Sweden; Escuela de Medicina y Ciencias de la Salud, Tecnologico de Monterrey, 14380, Mexico City, Mexico; Department of Medical Biochemistry and Biophysics, Karolinska Institutet, Solna SE-171 64, Sweden; Translational and Clinical Research Institute, Newcastle University, Newcastle upon Tyne NE4 5PL, UK; LUMC Genetic Small Vessel Disease Expert Center, Department of Clinical Genetics, Leiden University Medical Center, Leiden 2333 ZA, The Netherlands; Department of Cell and Molecular Biology, Karolinska Institutet, Solna SE-171 64, Sweden; Department of Neurobiology, Care Science and Society, Division of Neurogeriatrics, Karolinska Institutet, Solna SE-171 64, Sweden; Department of Neurobiology, Care Science and Society, Division of Neurogeriatrics, Karolinska Institutet, Solna SE-171 64, Sweden; Department of Neurobiology, Care Science and Society, Division of Neurogeriatrics, Karolinska Institutet, Solna SE-171 64, Sweden

**Keywords:** small vessel disease, vascular dementia, neurodegeneration, ischaemic stroke

## Abstract

Cerebral autosomal dominant arteriopathy with subcortical infarcts and leukoencephalopathy (CADASIL) is a small vessel disease caused by cysteine-altering *NOTCH3* gene variants, leading to vascular smooth muscle cell degeneration, compromised cerebral blood flow, subcortical ischaemic infarcts, cognitive decline and often ultimately vascular dementia. Little is known about the cellular and molecular effects downstream of the cerebral ischaemia in CADASIL, or whether brain regions known to be involved in dementia, such as the hippocampus, are particularly susceptible to such pathological downstream changes.

In this study, we used a humanized CADASIL mouse model harbouring the p.(Arg182Cys) variant (R182C-TgN3), post-mortem human CADASIL brain sections with four different *NOTCH3* gene variants and primary human cerebral vascular smooth muscle cells (VSMCs) harbouring the p.R133C *NOTCH3* variant as primary cellular models to characterize the properties and contribution of mutant VSMCs to cognitive impairment. To specifically evaluate neuronal, mitochondrial and neurovascular function, we performed *ex vivo* electrophysiology, immunohistochemistry [confocal and immunolabelling-enabled 3D imaging of solvent-cleared organs (iDISCO+) methods], western blotting, Seahorse assay, quantitative PCR and single-cell RNA sequencing.

In the CADASIL mice, hippocampal gamma oscillation patterns were impaired along with significant decreases in neuronal fibre length and aberrant neuronal morphology. The latter two phenotypes were also observed in post-mortem brain tissue from CADASIL patients. Consistent with these findings, we noted significantly lower levels of mitochondrial respiratory complexes in the CADASIL mouse hippocampus, isolated mouse brain vessels and primary human cerebral VSMCs. The human cerebral VSMCs exhibited reduced oxygen consumption rates leading to reduced ATP production as well as decreased glycolytic capacity in conjunction with increased pro-inflammatory gene expression, suggesting a broader impact on cellular energy metabolism and a neuroinflammatory process. In the CADASIL mice, we also observed extensive accumulation of the NOTCH3 extracellular domain in hippocampal vessels. Light sheet imaging with iDISCO+ clearing demonstrated substantial VSMC loss and reduced vessel density in the hippocampus at 9 months of age. Additionally, 3D imaging showed increased microglial attachment to vessels and enlargement of the size of the vessel-associated microglia in CADASIL mice. Single-cell RNA sequencing revealed a microglial subcluster expressing genes involved in mitochondrial respiration and inflammation.

Collectively, our results reveal how small vessel pathology in CADASIL leads to significant neuronal pathology in the hippocampus involving metabolic and neuroinflammatory changes and highlight the critical role of the neurovascular unit. Our findings pave the way for future research and potential therapeutic strategies.

## Teaser

Using human and mouse models, Shao *et al.* uncover hippocampal dysfunction in CADASIL, the most common monogenic form of small vessel disease. They demonstrate impaired hippocampal gamma oscillations alongside neuronal and mitochondrial dysfunction, vascular loss, and inflammation.

## Introduction

Cerebral autosomal dominant arteriopathy with subcortical infarcts and leukoencephalopathy (CADASIL) is the most common monogenic form of cerebral small vessel disease and is typically caused by cysteine-altering missense variants in the *NOTCH3* gene;^1^ for review see Coupland *et al*.^2^ CADASIL patients experience migraine with aura, subcortical ischaemic infarcts, mood disturbances and, in the advanced stages, varying degrees of vascular cognitive impairment (VCI).^3,4^ The incidence of CADASIL is estimated to be minimally 2–5:100.000, but the frequency of cysteine-altering *NOTCH3* variants has in public genomic databases been found to be considerably higher, approximately 300:100.000.^5^

**Figure 1:**
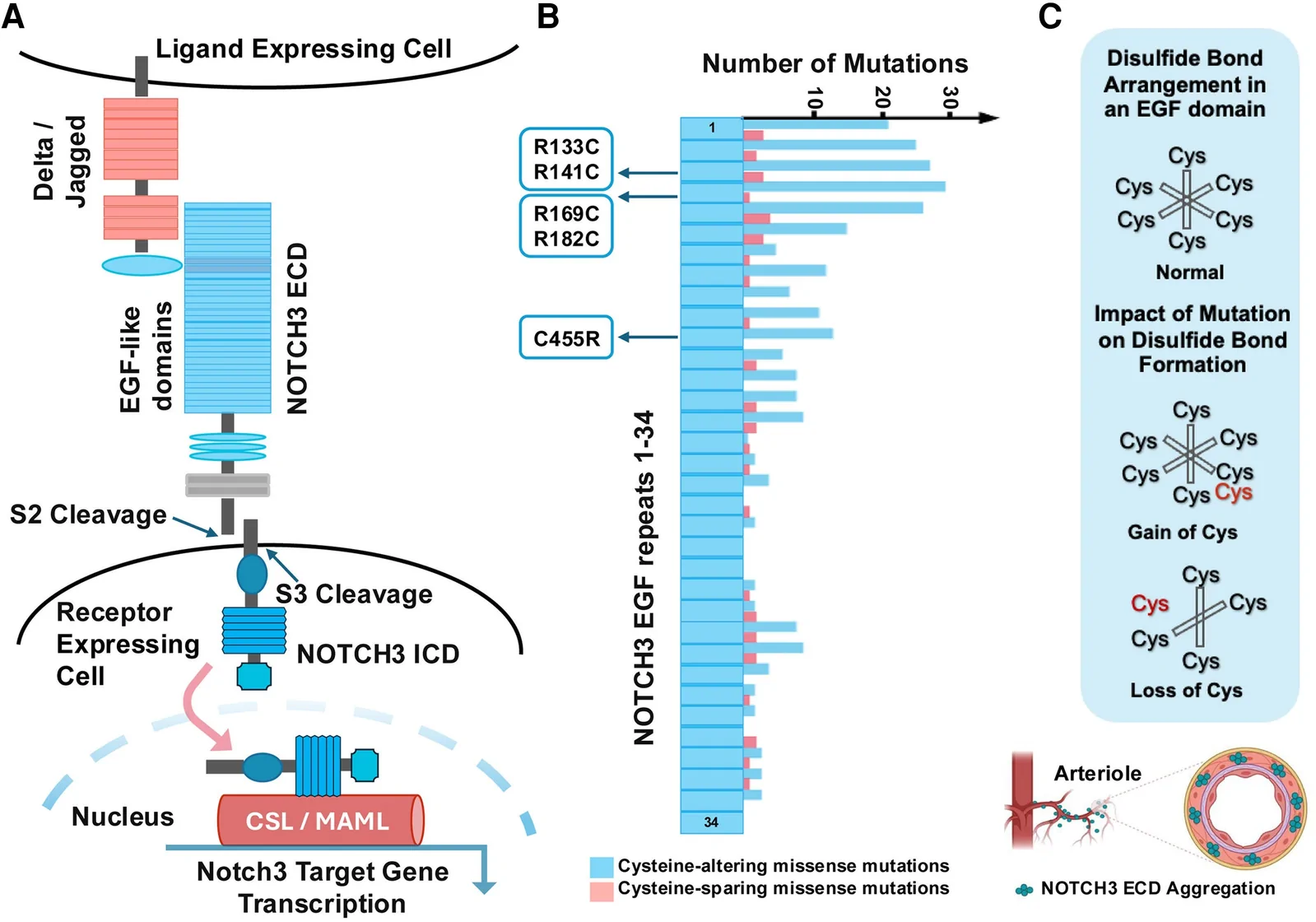
**The notch signalling pathway and CADASIL mutations in the NOTCH3 receptor.** (**A**) Schematic depiction of the Notch signalling pathway. (**B**) Schematic depiction of the distribution of CADASIL variants in the human NOTCH3 receptor and the localization of the mutations used in this study. (**C**) Visualization of the disulphide pairing of six cysteine (Cys) residues (normal) and gain or loss of one Cys residue and accumulation of NOTCH3 extracellular domain (NOTCH3 ECD) in the arteriole vessel wall. CADASIL = cerebral autosomal dominant arteriopathy with subcortical infarcts and leukoencephalopathy. This figure was created using BioRender. Shao, W. (2026) https://BioRender.com/lkarzmr.

The *NOTCH3* gene encodes a transmembrane receptor, which is activated by ligands of the Jagged or Delta-like type presented on a neighbouring cell (Fig. 1A).^6^ Ligand activation leads to two proteolytic cleavage events in the NOTCH3 receptor, ultimately releasing the C-terminal intracellular domain of the receptor (NOTCH3 ICD), which translocates into the nucleus. In the nucleus, NOTCH3 ICD interacts with the DNA-binding protein CSL (also known as RBP-J) and a third protein, MAML. The ternary NOTCH3 ICD/CSL/MAML complex regulates expression of Notch downstream genes (Fig. 1A). In CADASIL, missense mutations are found across the 34 epidermal growth factor (EGF)-like repeat (EGFr) domains in the extracellular domain of the NOTCH3 receptor (NOTCH3 ECD) (Fig. 1B).^1,7^ Notably, the vast majority of CADASIL variants affect a cysteine residue, either by introducing a novel cysteine residue or by losing one of the original six cysteine residues in an EGFr-domain (Fig. 1C). Both introduction and loss of a cysteine residue result in an unpaired cysteine, which may hinder the clearance of NOTCH3 ECD. In the human CADASIL brain vasculature, NOTCH3 ECD has indeed been shown to accumulate in the basal membrane surrounding vascular smooth muscle cells (VSMCs) and pericytes, the cell types that express NOTCH3 (Fig. 1C).^3,4^

While the accumulation of NOTCH3 ECD-containing aggregates around the VSMCs in the brain may explain the primary vascular phenotype, in which the NOTCH3-expressing VSMCs gradually succumb, leading to the arteriopathy and reduced cerebral blood flow causing subcortical ischaemic infarcts and cognitive decline. The hippocampus may play an important role in cognitive decline in CADASIL patients, as it has been shown that the number and volume of neurons in the hippocampus is reduced in CADASIL patient brains.^8^ Skin vessels also contain NOTCH3 aggregates in the vessel wall, and it has been shown that there is a significant reduction of nerve fibres in skin biopsies of CADASIL patients.^9^ Furthermore, disruptions of cortical-cortical and subcortical-cortical networks in CADASIL brains have been noted^10^ and magnetic resonance analysis has revealed early neuronal damage in CADASIL patients.^11^ Learning and hippocampal adult neurogenesis may also be affected in a CADASIL R169C mouse model,^12^ and physical exercise stimulated hippocampal neurogenesis in CADASIL mice harbouring the R169C mutation.^13^ However, despite these observations, relatively little is known about the role of the hippocampus and pathology at the neuronal molecular and cellular level.

In this article, we assess the neurovascular unit in CADASIL, using a combination of transgenic mice, analysis of post-mortem human CADASIL brains and primary cerebral human VSMCs. Notably, we observed an impaired hippocampal gamma oscillation pattern in the CADASIL R182C-TgN3 mouse model (hereafter referred to as CADASIL mice), which was accompanied by extensive reduction of neurite length and nerve fibre density, the latter was also observed in hippocampal sections from two post-mortem CADASIL patients with different *NOTCH3* variants. Furthermore, in CADASIL mice and human CADASIL VSMCs, we noted mitochondrial impairment. Finally, there was a dramatic loss of smooth muscle actin (SMA) coverage accompanied by an increased number of vasculature-interacting microglial cells in the CADASIL mice. Together, these findings suggest a strong connection between vascular dysfunction, microglial activation and neuronal impairment. This complex interplay sheds new light on the neuronal aspects of CADASIL and contributes to an improved understanding of the cognitive decline observed in CADASIL patients.

## Materials and methods

### Mouse models

The CADASIL R182C-TgN3 mice and the control mice, wild-type (WT)-TgN3, have previously been described by Rutten *et al*.^14^ The mice were housed at the Department of Comparative Medicine (KMB) at the Karolinska Institutet in Stockholm, Sweden. The mice were given food and water *ad libitum* and were kept in a 12-h light/dark cycle in enriched cages. All experimental procedures included 8–9-month-old mice of both sexes and were conducted in accordance with local regulations and rules and were approved by the Stockholm Animal Ethics Board (ethical permit no. 4433-2020 and 1113-2025), in line with the ARRIVE guidelines.

### Electrophysiology

WT-TgN3 and R182C-TgN3 male mice at 8 months of age were used for electrophysiology analysis (*n =* 3 mice per group). One animal per day was randomly selected and deeply anaesthetized using isofluorane before being sacrificed by decapitation. Electrophysiology was performed and analysed as described previously.^15^ Briefly, after brain extraction, we obtained ventral hippocampal horizontal dissections (350 μm thick) using a Leica VT1200S vibratome. *Ex vivo* gamma oscillations were elicited by kainic acid (KA) in the CA3 area of the hippocampus and gamma-spike coupling of cornu ammonis 3 (CA3) pyramidal neurons (PCs) and fast-spiking interneurons (FSIs), and the experiment was performed in a submerged chamber set-up. Gamma oscillations were analysed using the pCLAMP 10.4 software and Axograph software. Spike-gamma coupling was analysed with custom-made routines in MATLAB.

### Cell culture

CADASIL patient-derived cerebral arterial VSMC (VSMC^R133C^) as well as control VSMC (VSMC^WT^) cell lines were established from post-mortem subarachnoidal branches of cerebral arteries^16^ and maintained in Dulbecco's Modified Eagle's Medium (DMEM)-F12, supplemented with 10% fetal bovine serum (FBS), 1% penicillin-streptomycin and 1% L-glutamine, 1% smooth muscle growth supplement. The cell lines were kept in an incubator with 5% CO_2_ at 37°C.

### Western blotting

Samples of mouse hippocampus or cortex (*n =* 4 per genotype) were freshly harvested and lysed in radio immunoprecipitation assay (RIPA) lysis buffer with a phosphatase and protease inhibitor. Human cerebral VSMCs were also lysed in RIPA lysis buffer. The isolation and purification of mouse brain blood vessels were performed as previously described.^17^ The purified vessels were lysed using a lysis buffer containing 0.5 M Tris-Cl, 4% sodium dodecyl sulfate (SDS), 1% bromophenol blue, 50% glycerol and 20 mM L-dithiothreitol (DTT). The total protein extracts were denatured at 95°C and electrophoresed on 4%–12% SDS gels using NuPAGE™ MOPS running buffer. For analysis of oxidative phosphorylation (OxPhos) proteins, the lysates were denatured at 55°C. After being transferred to a polyvinylidene difluoride (PVDF) membrane, the membranes were blocked in the blocking buffer for 2 h, followed by incubation with the primary antibody overnight at 4°C. The membranes were then washed with TBST (Tris-buffered saline containing 0.1% Tween 20), followed by the secondary antibody incubation for 2 h at room temperature. After washing with TBST, the membranes were scanned using an Odyssey fluorescent scanner (LICOR Biosciences). The antibodies used for the analysis were OxPhos rodent western blotting (WB) antibody cocktail, neurofilament heavy chain (NFH), postsynaptic density protein 95 (PSD95), synaptophysin (SYP), ubiquitin carboxyl-terminal esterase L1 (UCHL1/PGP9.5), GAPDH, β-actin and actin (Supplementary Table 1).

### Quantitative real-time PCR analysis

Total RNA was extracted from VSMC^R133C^ and control VSMC cell lines using the RNeasy mini kit. The quantity of RNA was measured using NanoDrop, and cDNA was prepared with the Maxima First Strand cDNA Synthesis Kit using 300 ng of RNA. Real-time PCR analysis was then performed using *N'*,*N'*-dimethyl-*N*-[4-[(E)-(3-methyl-1,3-benzothiazol-2-ylidene)methyl]-1-phenylquinolin-1-ium-2-yl]-*N*-propylpropane-1,3-diamine (SYBR) Green master mix on the Applied Biosystems 7500 Fast Real-Time PCR System, following the manufacturer’s protocol. Primers used for qPCR are listed in Supplementary Table 2. Relative mRNA expression levels are normalized to the average housekeeping RNA levels of β-actin using the 2^−ΔΔCt^ method.^18^

### Immunofluorescence analysis

After complete anaesthesia with pentobarbital sodium, 9-month-old mouse brains (*n* = 5) were perfused with PBS and 4% paraformaldehyde (PFA) before being harvested and post-fixed for 6 h at room temperature. Vibratome sections of 40-mm thickness brain slices were then permeabilized and blocked with blocking buffer containing 2% bovine serum albumin (BSA), 5% donkey serum and 0.75% triton overnight at 4°C. Brain floating slices were incubated with primary antibodies overnight at 4°C, the antibodies used were mouse NOTCH3 1E4, CD31, NFH, UCHL1, and NeuN, IBA1, followed by incubation with the corresponding fluorescence secondary antibodies (Supplementary Table 1). For sections labelled with 4′,6-diamidino-2-phenylindole (DAPI), the sections were stained for 15 min at room temperature after incubation with secondary antibody. The stained sections were imaged using a Zeiss LSM 880 microscope. Whole hippocampus images were acquired by the tile scan function. Z-stacks of images were processed using ImageJ software (Version 2.0.0) (Fiji distribution).

### Immunohistochemistry analysis

Immunohistochemistry was performed as previously described.^17^ In brief, human brain paraffin-embedded sections were deparaffinized and antigen retrieved with Diva decloaker. Endogenous peroxidases were blocked with Peroxidazed 1 for 5 min. Sections were then incubated with a primary antibody targeting UCHL1 or NFH, followed by incubation with MACH3 Mouse HRP-Polymer Detection. After dehydration, the sections were mounted, and images were taken on a Nikon Eclipse E800 microscope. The quantification of nerve fibre length was performed using NIS-Elements software from Nikon Instruments Inc. The human brain paraffin-embedded sections were provided by the Newcastle Brain Tissue Resource, A. Louvi at Yale School of Medicine and H. Kalimo at the University of Helsinki (Supplementary Table 3), and the experiments were conducted in accordance with local regulations and rules and were approved by the Swedish Ethical Review Authority (#2024-03239-01).

### Brain tissue clearing and 3D imaging by light sheet fluorescent microscopy

The immunolabelling-enabled 3D imaging of solvent-cleared organs (iDISCO+) tissue-clearing protocol was utilized to render the entire mouse brain transparent for high-resolution 3D imaging.^19^ Briefly, brain tissue from a 9-month-old brain (*n =* 5 per genotype) was first perfused with PBS and 4% PFA, and subsequently dehydrated using 33% dichloromethane (DCM) to remove lipids. A bleaching step with 5% hydrogen peroxide in methanol was applied to minimize endogenous pigmentation. Following rehydration, the tissue was blocked to prevent non-specific antibody binding and then immunolabelled with primary and secondary antibodies. A final clearing step was performed using a refractive index-matching solution of dibenzyl ether (DBE) to achieve whole brain tissue transparency. For specific visualization of blood vessels, endothelial cells were labelled with CD31 antibody, while arteries were identified using anti-alpha smooth muscle actin antibody (α-SMA). IBA1 was used to label microglia. Imaging of the hippocampus was conducted using a LaVision Light Sheet Microscope (LaVision Biotec) from a horizontal view: for images labelled with α-SMA, magnification is ×6.4; for images co-labelled with CD31 and IBA1, magnification is ×8, the total thickness of the hippocampus region is 1000 μm and the step size is 2 μm. The overview of arteries in the hippocampus, magnification is ×3.2, the total thickness of the hippocampus region is 2500 μm and the step size is 3 μm. After acquiring 3D images, segmentation and vessel quantification in the hippocampus area was performed using Imaris software (version 10.2.0) to facilitate detailed quantitative and structural analysis.

### Seahorse analysis

The oxygen consumption rate (OCR), an indicator of mitochondrial respiration, and extracellular acidification rate (ECAR), which is proportional to glycolysis, were assessed in real-time live-cell extracellular flux analysis using the Seahorse XF96 analyser (Agilent) according to the manufacturer’s instructions. Human cerebral VSMCs with or without the CADASIL NOTCH3 R133C mutation were plated into Seahorse XF96 plates at a density of 26 000 cells per well. Before the experiment, cell culture media was replaced by DMEM supplemented with 0.23 mM sodium pyruvate, 2 mM L-glutamine and 10 mM glucose (pH = 7.4) (for OCR) or DMEM supplemented with 2 mM L-glutamine (pH 7.4) (for ECAR). Both OCR and ECAR were then measured at baseline. Subsequently, cells were stimulated by serial injection of 1 µM oligomycin A, 1.5 µM carbonyl cyanide-p-(trifluoromethoxy) phenylhydrazone (FCCP) and 0.5 µM rotenone plus 0.5 µM antimycin A for OCR; or 10 mM glucose, 1 µM oligomycin A and 50 mM 2-deoxy-D-glucose for ECAR. OCR results were obtained using the Seahorse XF Cell Mito Stress report generator while ECAR data was automatically analysed by the Seahorse XF Glycolysis Stress Test summary report, both provided by Agilent’s Wave 2.6.1 software. OCR values expressed in pmol O_2_/min and ECAR values expressed in mpH/min were normalized to protein content using the Pierce BCA Protein Assay Kit.

### Transmission electron microscopy

After anaesthesia with pentobarbital sodium, WT-TgN3 (*n* = 2) and R182C-TgN3 (*n* = 2) mice were perfused with PBS and 2.5% glutaraldehyde and 1% formaldehyde in 0.1 M Sorensen phosphate buffer. The hippocampus was isolated and immersion fixed in the same buffer at +4°C. The fixed samples were rinsed in 0.1 M phosphate buffer followed by post-fixation in 2% osmium tetroxide in 0.1 M phosphate buffer, pH 7.4 at 4°C for 2 h. After stepwise dehydration in ethanol and *en bloc* staining using 0.5% uranyl acetate (UAc), the samples were acetone dehydrated prior to resin infiltration and finally embedded in LX-112 (Ladd Research). Ultrathin sections (∼80–100 nm) were prepared using an EM UC7 (Leica) and transferred onto formvar slot grids and contrasted with uranyl acetate followed by lead citrate. The grids were examined in a HT7700 transmission electron microscope (Hitachi High-Technologies) at 80 kV and digital images were acquired using a 2kx2k Veleta CCD camera (Olympus Soft Imaging Solutions).

### Single-cell capture

Cerebrovascular cells from WT-TgN3 (*n* = 5) and R182C-TgN3 (*n* = 6) mice of 6 to 9 months of age were isolated using CD31 panning according to a previously published protocol.^20^ Cells were loaded in the 10× Genomics Chromium controller using the Next Gem Single Cell 3’ Reagent kit v3.1 (Dual Index) aiming for 5000 cells per sample. cDNA and libraries were generated according to manufacturer’s instructions (protocol: CG000315). Samples were sequenced using Next seq 2000 P3 100 cycles flow cells aiming for 40 000 reads/cell.

### Single-cell RNA sequencing data analysis

Sequencing flow cells were demultiplexed and aligned using the Cellranger Pipeline v7.0.0 (10× Genomics). The transcriptome used for mapping was mm10 from 2020 with the human *NOTCH3* gene manually added. Data was analysed using the Seurat pipeline v.5.0.2 (doi:10.1038/nbt.4096) for R v.4.3.1. Quality control (QC) and annotation are described in a previous publication.^20^ Isolation of the microglial supercluster was done based on cluster annotation and followed by further quality control which led to the exclusion of one control sample due to a highly polarized landscape of microglia which was not observed in the remaining samples (neither CADASIL nor control). Pseudo bulk differentially expressed gene (DEG) analysis was carried out on data where ribosomal genes, mitochondrial genes, Gm genes and Rik genes were removed using AggregateExpression followed by FindMarkers with DESeq2 test between WT-TgN3 and R182C-TgN3 with Seurat pipeline. Adjusted *P*-values *<* 0.05 were considered significant. Marker genes for the subclusters were used for gene ontology (GO) analysis using ‘clusterProfiler’ v.4.8.3 also for R. All three sub-ontologies, biological process (BP), cellular component (CC) and molecular function (MF), were included in the enrichment analysis. No original code was used for the single-cell (sc)RNA sequencing (scRNAseq) analysis.

### Statistical analysis

All statistical analyses were conducted using GraphPad Prism (Version 8.0 and 10.0), ensuring that assumptions of normality and homogeneity of variance were checked prior to conducting the *t*-tests. An independent samples *t*-test was used to evaluate whether there were statistically significant differences in the measured variables. Results are reported as mean ± standard error of the mean. The level of statistical significance was set at *P <* 0.05. The *P*-values for the single-cell analysis were corrected with the in Seurat built-in correction method for the Wilcoxon Rank Sum test.

## Results

### Extensive loss of neuronal fibres in the CADASIL mouse model and human post-mortem CADASIL brains

**Figure 2:**
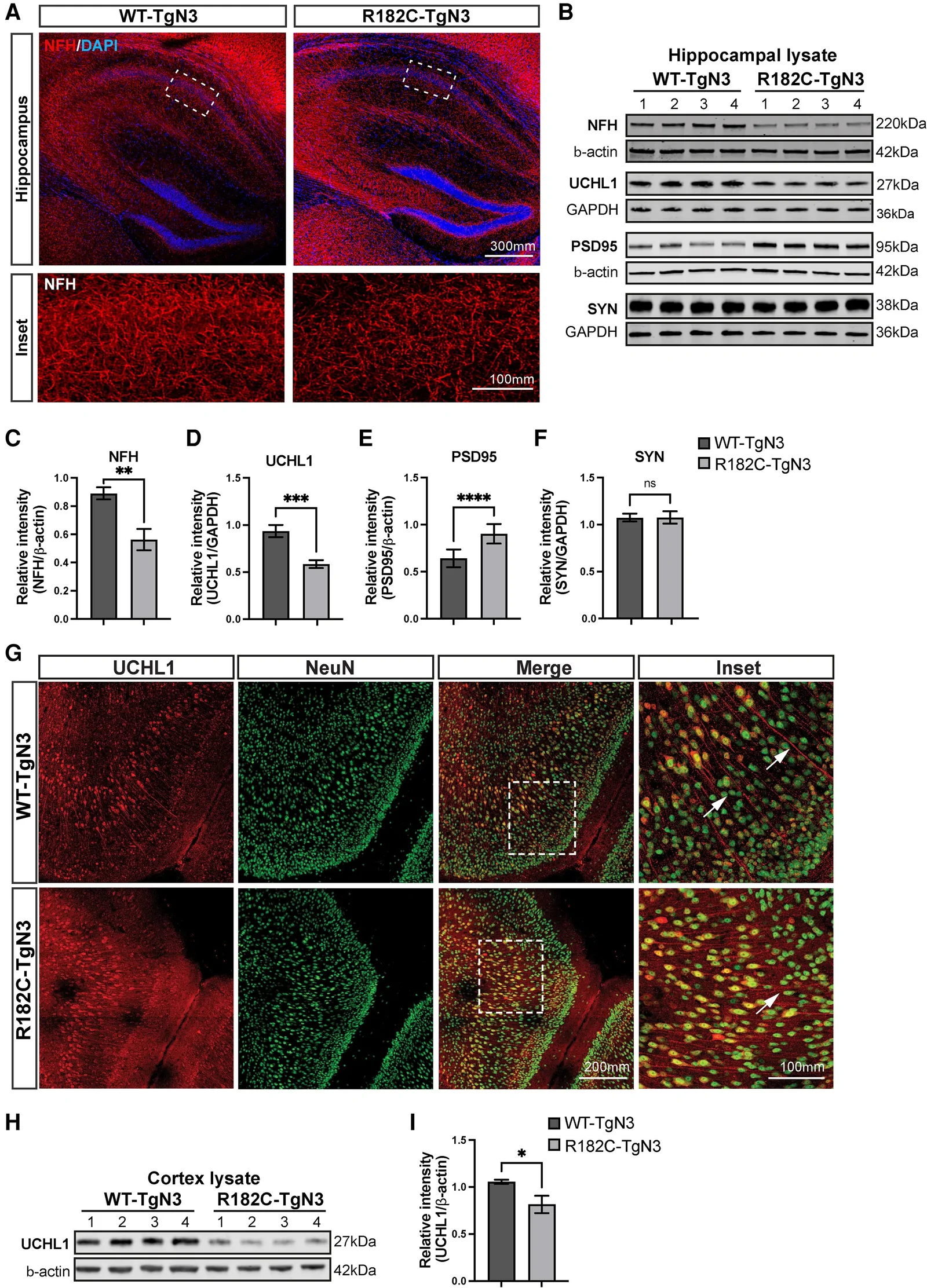
**Loss of hippocampal neuronal fibres and altered neuronal morphology in CADASIL mice.** (**A**) Confocal images of neurofilament heavy chain (NFH) and 4′,6-diamidino-2-phenylindole (DAPI) staining in the hippocampus of R182C-TgN3 and WT-TgN3 mice, as indicated. The rectangular dashed boxes mark the regions of interest, which are shown at higher magnification in the inset. (**B**) Western blot analysis of NFH, Ubiquitin C-terminal hydrolase L1 (UCHL1), Post-Synaptic Density Protein 95 (PSD95) and Synaptophysin (SYN) expression in hippocampal lysate of R182C-TgN3 and WT-TgN3 mice. GAPDH and β-actin were used as loading controls (*n* = 4 per genotype). (**C**–**F**) Relative quantification is depicted in the bar graphs, as indicated. (**G**) Confocal images of UCHL1 and NeuN staining in the cortex of 9-month-old R182C-TgN3 and WT-TgN3 mice, as indicated. The rectangular dashed boxes mark the regions of interest, which are shown at higher magnification in the inset. Nerve fibres are depicted (white arrows). (**H**) Western blot analysis of UCHL1 expression in cortical lysates from R182C-TgN3 and WT-TgN3 mice. β-actin was used as loading controls (*n* = 4 per genotype). Relative quantification is depicted in the bar graphs, as indicated. (**C**–**F** and **I**) All representative western blot results are from three technical replicates. The cropped western blot images in this figure are derived from the original blots shown in the Supplementary material. Bar graphs represent the mean ± standard error of the mean. An unpaired two-tailed Student’s *t*-test was used for statistical analysis. *n* = 3 technical replicates, *P < 0.05, **P < 0.01, ***P < 0.001, ****P < 0.0001, ns = non-significant. CADASIL = cerebral autosomal dominant arteriopathy with subcortical infarcts and leukoencephalopathy; WT = wild-type.

The hippocampus plays a central role in several cognitive aspects, including learning and memory^21^ and to explore the underlying basis for the cognitive decline in CADASIL patients, we first examined whether the hippocampal neuronal fibres in the CADASIL mice were affected. The CADASIL mouse model displays several features of CADASIL, including accumulation in brain arterioles of the NOTCH3 ECD and granular osmiophilic dense material (GOM),^14,22^ the latter feature is also a hallmark of CADASIL pathology.^3^ As a control, we used a transgenic mouse, WT-TgN3 (hereafter referred to as control mice), which expresses wild-type human NOTCH3 at similar levels as endogenous mouse Notch3 receptor.^14^ In hippocampal sections from the CADASIL mice we noted a significant reduction of NFH immunoreactivity, which provides structural support to neurons,^23^ compared with the control mice and the NFH-positive fibres were also more disorganized in the CADASIL mice (Fig. 2A). Further, western blotting analysis of hippocampal lysates, to quantify neuronal, lysosomal, and synaptic markers from CADASIL and control mice, showed reduced levels of NFH in CADASIL mice compared with controls (Fig. 2B). In addition, the level of ubiquitin C-terminal hydrolyze L1 (UCHL1), a deubiquitinase serving as a pan-marker for neurons,^24^ was reduced. Conversely, the levels of postsynaptic density protein-95 (PSD95),^25^ a marker for excitatory synapses, were elevated. However, we could not detect any differences in synaptophysin (SYN) immunoreactivity (Fig. 2C–F). Shortened neuronal fibres were not only observed in the hippocampus, but were also evident in the prefrontal cortical region of the CADASIL mice, determined by immunostaining with UCHL1 and NeuN (another pan-neuronal marker staining the nuclei)^26^ (Fig. 2G). Assessment of UCHL1 protein level in the prefrontal cortical region by western blot analysis (Fig. 2H and I) similarly showed significantly reduced expression of UCHL1.

**Figure 3:**
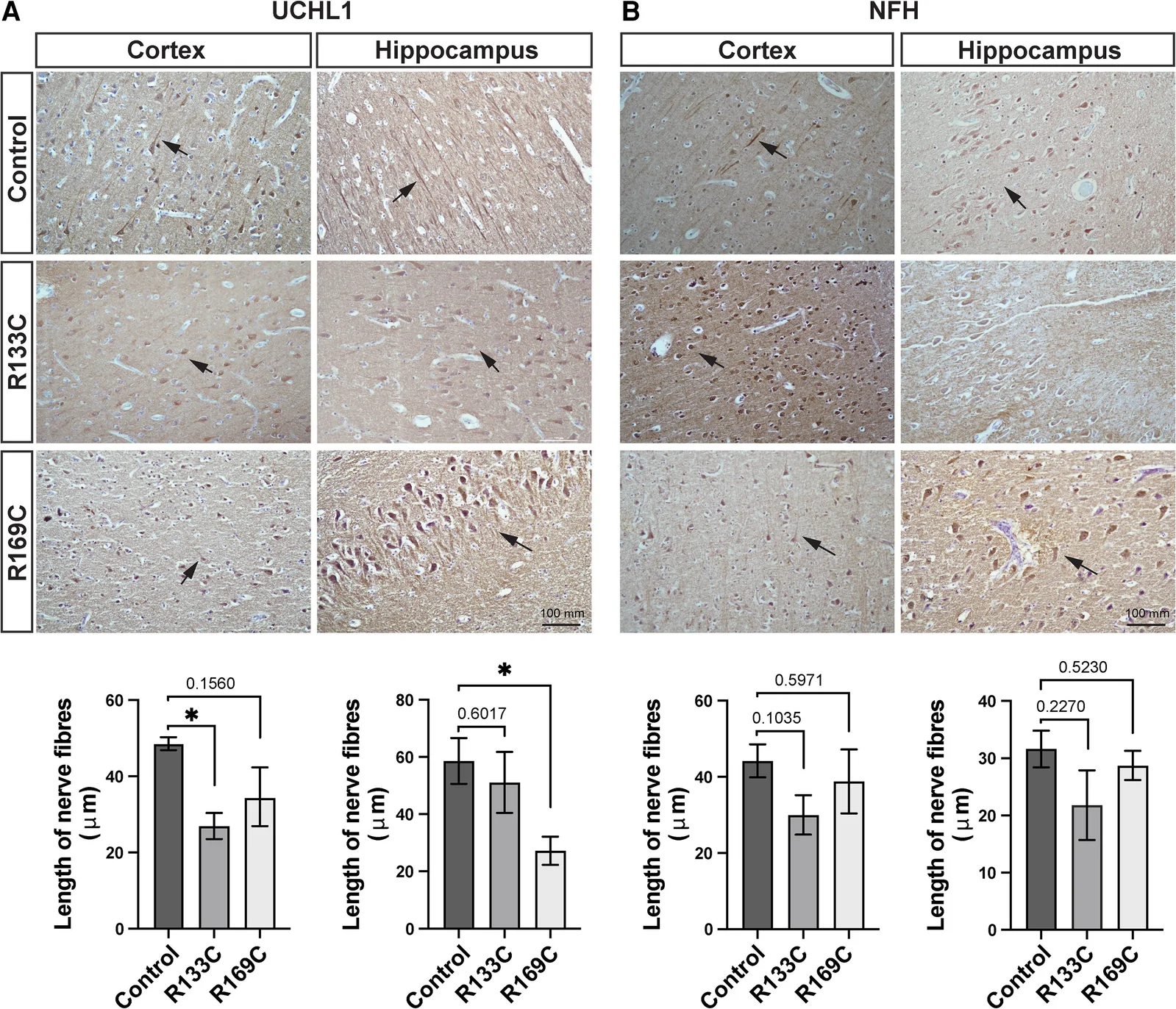
**Loss of neuronal fibres and altered neuronal morphology in human CADASIL brains.** Immunohistochemical analysis (20×) of Ubiquitin C-terminal hydrolase L1 (UCHL1) (**A**) and neurofilament heavy chain (NFH) (**B**) staining in histological sections of the cortex (*left*) and hippocampus (*right*) from control and CADASIL patients with p.R133C and p.R169C *NOTCH3* variants, for more details of the clinical data, see Supplementary Table 3. Sections were counterstained with haematoxylin (blue), the arrows depict neuronal fibres. The *bottom* panels present the quantifications, measured in three random areas per brain region. The mean fibre length was used for statistical analysis. The values represent the mean ± standard error of the mean using an unpaired two-tailed Student’s *t*-test. *n* = 3, *P < 0.05. CADASIL = cerebral autosomal dominant arteriopathy with subcortical infarcts and leukoencephalopathy.

In order to understand to what extent these findings in the CADASIL mice translate to human CADASIL patients, we investigated the presence and amount of nerve fibres in post-mortem human CADASIL brain tissue. We analysed the neuronal fibres in histological sections from post-mortem prefrontal cortical and hippocampal brain tissue from two patients, each carrying a different *NOTCH3* variant (p.R133C and p.R169C, respectively). Analysis of the distribution of UCHL1 by immunohistochemistry revealed that the length of UCHL1+ nerve fibres was reduced in the two CADASIL patient brains (Fig. 3A). In addition, an aberrant neuronal morphology was revealed, with neurons showing a more runted morphology, accompanied by loss of nerve fibres (Fig. 3A). Immunostaining with NFH produced a similar trend: there was a reduced length of nerve fibres in the cortical and hippocampal sections from the two CADASIL patients, coupled with a runted neuronal morphology (Fig. 3B). These findings were confirmed in three additional post-mortem cortical brain tissues with p.R133C, p.R169C and p.C455R mutations using UCHL1 and NFH (Supplementary Fig. 1A and B). In sum, these results reveal extensive neuronal fibre loss and altered neuronal morphology in the human CADASIL brain and a CADASIL mouse model.

### Impaired hippocampal gamma oscillations pattern in CADASIL mice

**Figure 4:**
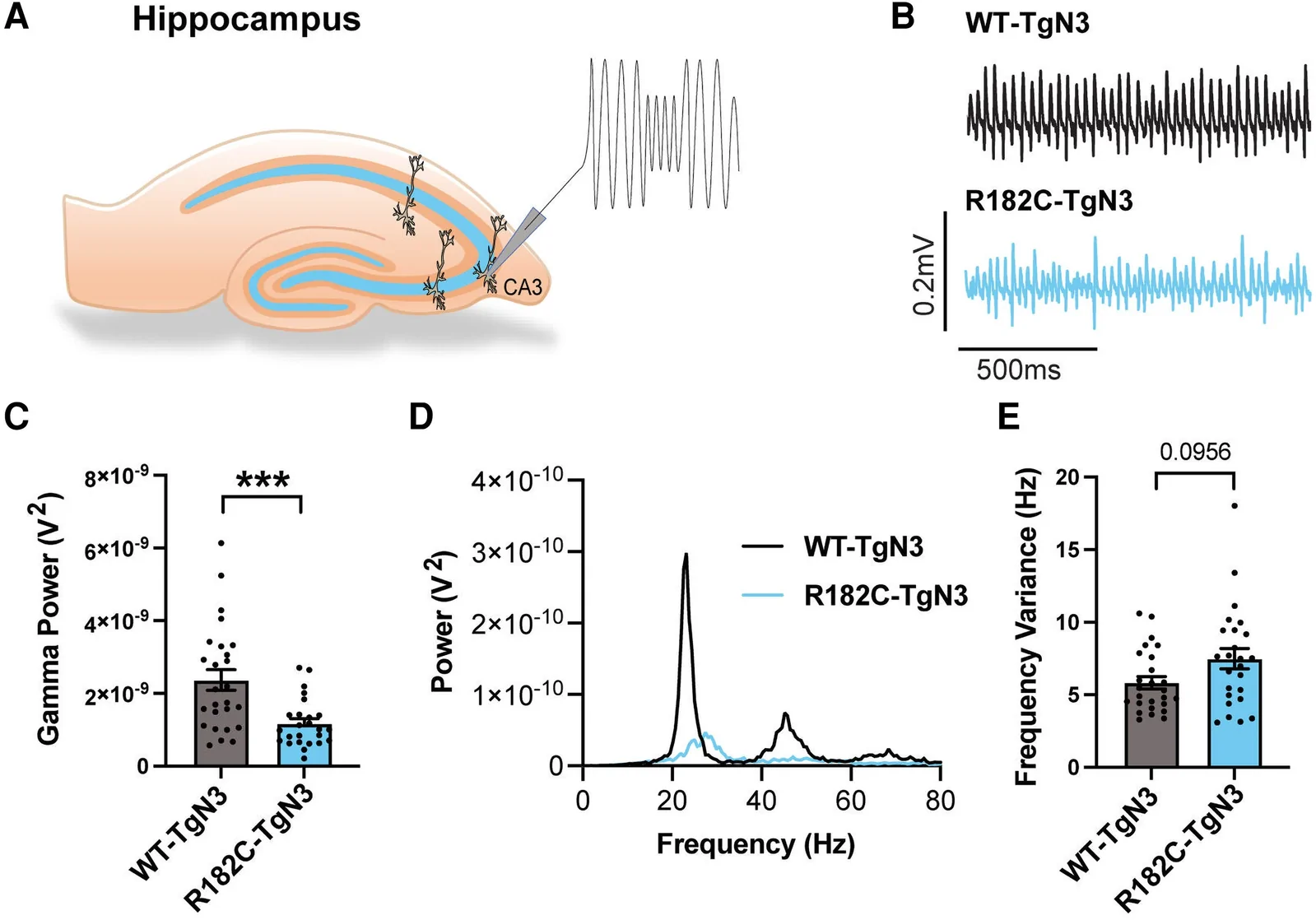
**Impairment in the hippocampal gamma oscillatory network in CADASIL mice.** (**A**) Schematic depiction of gamma oscillation in the hippocampal CA3 region. (**B**) Representative local field potential (LFP) traces from kainic acid (KA)-induced gamma oscillations in 9-month-old WT-TgN3 and R182C-TgN3 mice. (**C**) Bar graph of gamma power from the experimental conditions described in **A** (Mann–Whitney test, ***P < 0.001). (**D**) Representative power spectrum from the experimental conditions described in **A**. (**E**) Bar graph of frequency variance from the experimental conditions described in **A** (Mann–Whitney test). The number of animals used in the experiment is presented as dots in the bar graphs. CADASIL = cerebral autosomal dominant arteriopathy with subcortical infarcts and leukoencephalopathy; WT = wild-type.

We next sought to explore whether the shortened neuronal fibres observed in the hippocampus of the CADASIL mice directly affected the neuronal activity in the hippocampus. To this end, we analysed the gamma oscillations activity. Gamma oscillations are high-frequency brain waves (30–100 Hz),^27^ which have been associated with neurodegeneration in diseases such as schizophrenia and Alzheimer’s disease (AD). More specifically, gamma oscillations result from the rhythmic synchronization of action potentials generated by excitatory pyramidal cells (PCs) and inhibitory fast-spiking interneurons (FSNs), leading to rhythmic postsynaptic currents.^27^ Gamma oscillations coordinate pre- and postsynaptic firing and may be involved in memory formation.^28^ We analysed the gamma oscillation patterns in the CA3 of CADASIL and control mice (Fig. 4A). There was a significant decrease in gamma oscillation power in the CADASIL mice as compared with controls (Fig. 4B and C). Additionally, we observed increasingly disordered rhythms, evidenced by a further increase in frequency variance in CADASIL mice (Fig. 4D and E). Next, we evaluated the cellular activity of the network components in the gamma oscillations. Single-cell patch clamp recording PCs and inhibitory FSN were carried out in the CA3 region of the hippocampus concomitantly with ongoing gamma oscillations. We found that both cellular populations had similar membrane potential and excitatory input (Supplementary Fig. 2A–H). Our results indicate that the CADASIL mouse model exhibits impaired gamma oscillation pattern and gamma power.

### Impairment of mitochondria in hippocampus and brain vessels of CADASIL mice and human CADASIL VSMCs

**Figure 5:**
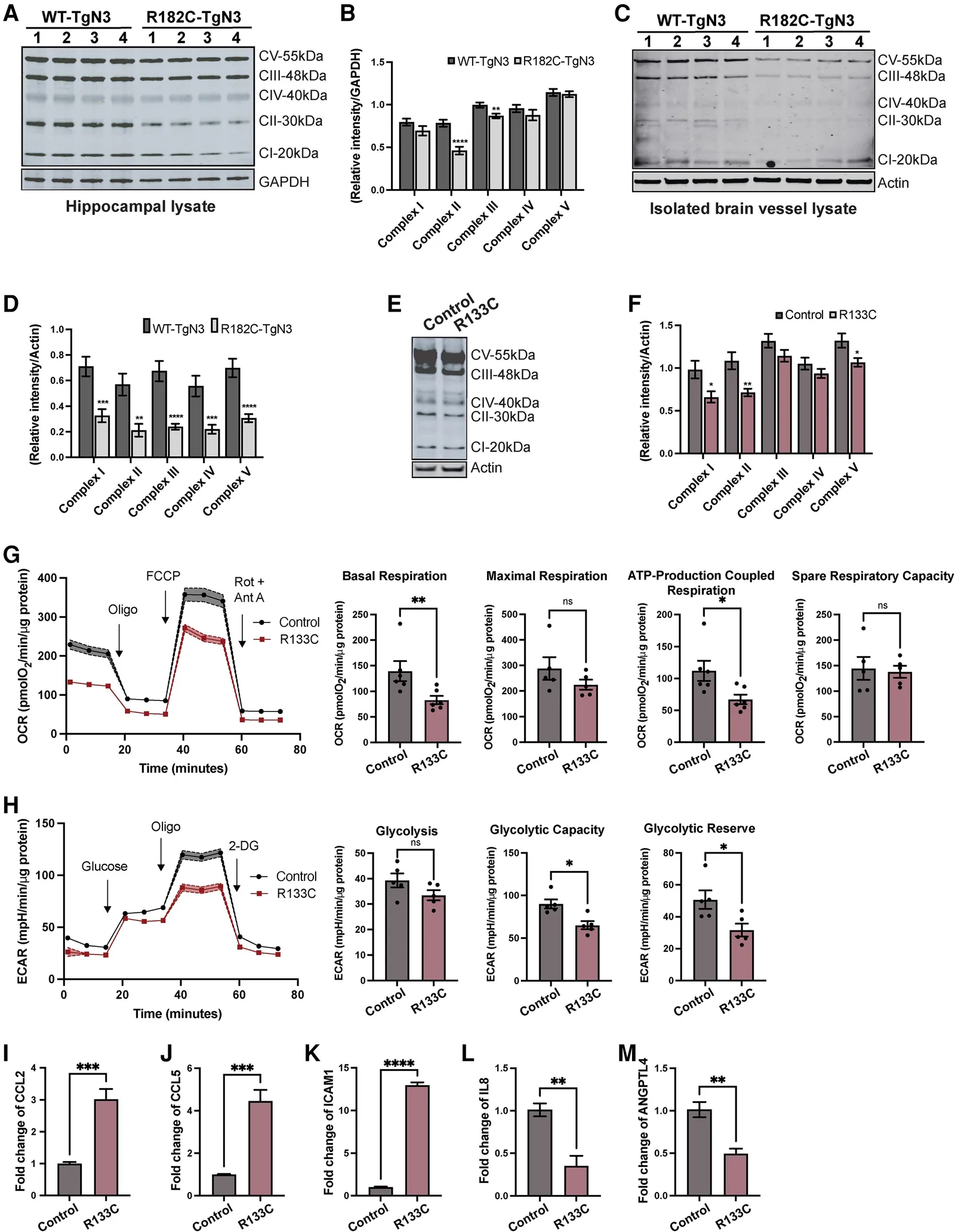
**Mitochondrial impairment in CADASIL mice and human VSMCs accompanied by elevated expression of inflammation markers.** (**A**) Representative immunoblot of OxPhos from hippocampal homogenates of 9-month-old mice as indicated (*n* = 4 per genotype). GAPDH was used as a loading control. (**B**) Relative quantification of each complex is depicted in the bar graphs, as indicated. (**C**) Representative immunoblot of OxPhos in isolated cerebral vessels from 9-month-old mice, as indicated (*n* = 4 per genotype). Actin was used as a loading control. (**D**) Relative quantification of each complex is depicted in the bar graphs, as indicated. (**E**) Representative western blot analysis of OxPhos of human control and R133C mutant VSMCs, actin was used as a loading control. Cropped western blot images in this figure are derived from the original blots shown in the Supplementary material. (**F**) Relative quantification of each complex is depicted in the bar graphs, as indicated (*n =* 4 as technical replicate). All bar graphs are presented as mean ± standard error of the mean (SEM). An unpaired two-tailed Student’s *t*-test was used for statistical analysis, *n* = 3 technical replicates. (**G** and **H**) Metabolic analysis of control and R133C mutant VSMC cells using a Seahorse XF96 analyser. (**G**) Oxygen consumption rate (OCR) to assess mitochondrial respiration and (**H**) extracellular acidification rate (ECAR) measured glycolysis. Graphs summarize average OCR and ECAR values for four replicates, Basal respiration; maximal respiration; ATP-production coupled respiration; spare respiratory capacity; glycolysis (after glucose addition); glycolytic capacity (after oligomycin); glycolytic reserve. All the bar graphs are presented as mean ± SEM. An unpaired Mann–Whitney test was used for statistical analysis (*n* = 5 as technical replicate). (**I**–**M)** Quantitative real-time PCR analysis of pro-inflammatory genes *CCL2* (**I**), *CCL5* (**J**) and *ICAM1* (**K**), and (**L** and **M**) anti-inflammatory genes *IL-8* (**L**) and *ANGPTL4* (**M**) in control and R133C mutant VSMC cells (*n* = 4 technical replicates). Bar graphs are presented as mean ± SEM. An unpaired two-tailed Student’s *t*-test was used for statistical analysis. **P < 0.01, ***P < 0.001, ****P < 0.0001.CADASIL = cerebral autosomal dominant arteriopathy with subcortical infarcts and leukoencephalopathy; OxPhos = oxidative phosphorylation; VSMC = vascular smooth muscle cell; WT = wild-type.

Synaptic transmission and, particularly, fast neuronal network oscillations, are associated with high energy demand and require rapid adaptation of oxidative energy metabolism.^15^ One possible explanation for the neuronal dysfunction and altered gamma oscillation patterns in the hippocampus of the CADASIL mice would be impaired mitochondrial function, and we therefore assessed levels of key proteins in each of the five respiratory complexes (CI–CV). These are inner mitochondrial membrane (IMM)-embedded protein complexes, and the first four complexes (CI–CIV) are part of the electron transport chain whose main role is to generate an electrochemical proton gradient across the IMM. Complex V (CV) is an ATP synthase that utilizes the proton gradient to generate ATP from adenosine diphosphate (ADP).^29^ Western blot analysis of hippocampal homogenates from the 9-month-old CADASIL and control mice revealed that the levels of complex II and complex III were reduced in the CADASIL mice, indicating that mitochondrial function is impaired in the CADASIL mice (Fig. 5A and B).

To understand the cellular origin of the impaired mitochondria, we performed a similar analysis on isolated brain vessels and found an impairment in all five respiratory complexes in the CADASIL mice compared with control mice (Fig. 5C and D). Moreover, in the human cerebral VSMCs harbouring the *NOTCH3* p.R133C CADASIL variant, the levels of the complexes I, II and V were also reduced compared with control VSMCs (Fig. 5E and F), suggesting complex V-dependent ATP defects.

To gain insight into the cellular metabolism of VSMCs in response to various stimuli or stressors, we performed Seahorse analysis to assess mitochondrial respiration and glycolysis. There was a significant reduction in basal respiration and ATP production in the CADASIL VSMCs, which aligns with decreased complex I/II and complex III, respectively, indicating mitochondrial impairments even in the absence of stress stimulus. To understand whether CADASIL VSMCs shift their metabolism from oxidative phosphorylation (OxPhos) to glycolysis, we blocked mitochondrial complex V with oligomycin and evaluated glycolysis as a compensatory response to mitochondrial deficits. While both VSMC genotypes respond similarly to glucose, CADASIL VSMCs showed deficient glycolytic capacity and reserve under mitochondrial inhibition (Fig. 5G and H). These data suggest that CADASIL VSMCs are impaired in the two major pathways for energy production. Since damaged mitochondria can lead to neuroinflammatory processes,^30^ we assessed some common pro-inflammatory genes in the human CADASIL VSMCs by qPCR and observed increased expression of the *CCL2, CCL5* and *ICAM1* genes (Fig. 5I–K), while the expression of the anti-inflammatory genes *IL-8* and angiogenic gene *ANGPTL4* conversely was reduced (Fig. 5L and M). Overall, these data indicate that mitochondrial dysfunction is observed in the CADASIL mouse model and the human CADASIL VSMCs, which may lead to neuroinflammation and be correlated to the observed neuronal phenotype in CADASIL mice and CADASIL patients.

### Loss of vascular smooth muscle cells in the hippocampal vasculature of CADASIL mice

**Figure 6:**
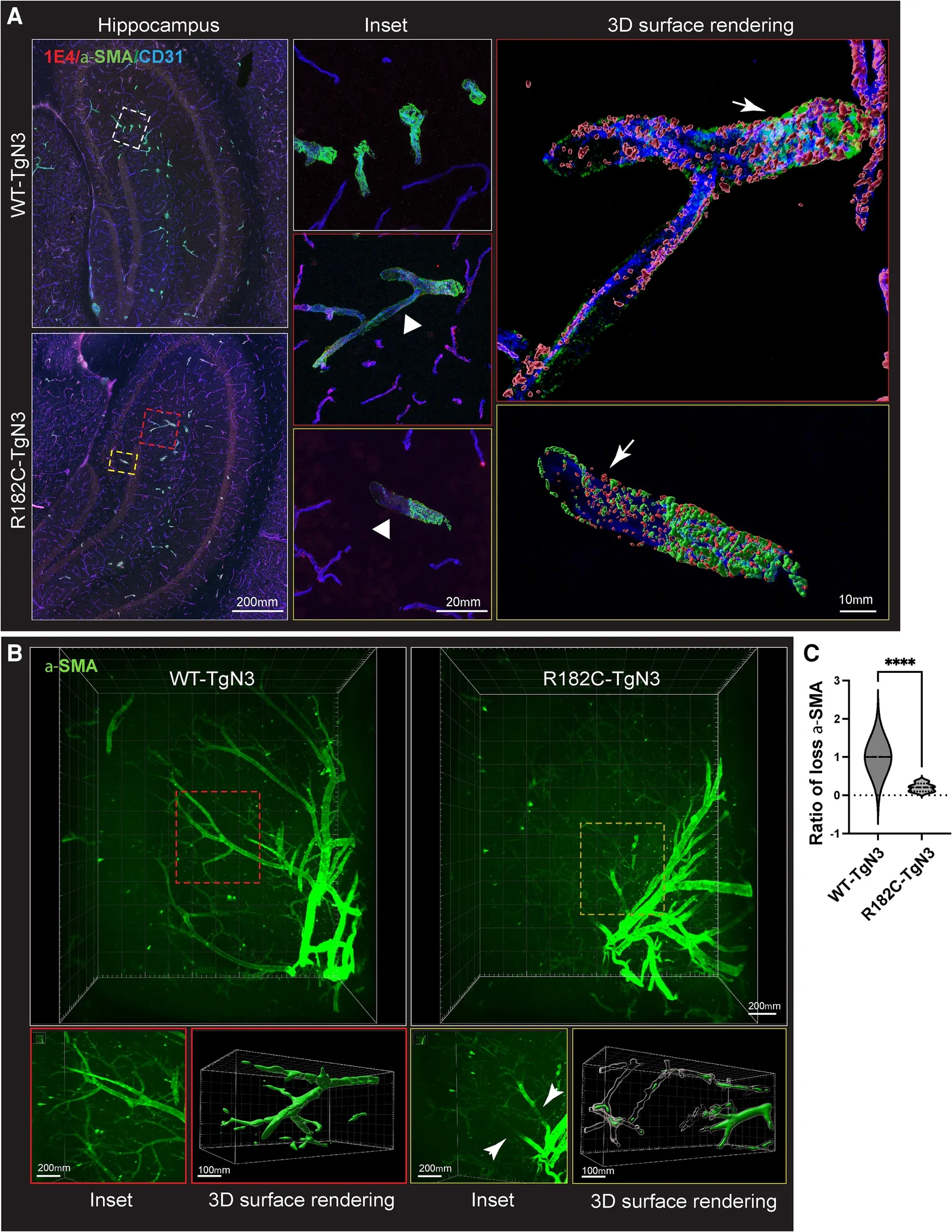
**NOTCH3 extracellular domain accumulation and VSMC loss in hippocampal vessels in CADASIL mice.** (**A**) Confocal images of the hippocampal region labelled with NOTCH3 extracellular domain (NOTCH3 ECD) (1E4 antibody, red), smooth muscle actin (SMA) (α-SMA antibody, green) and endothelial cells (CD31 antibody, blue) on sections from 9-month-old R182C-TgN3 and WT-TgN3 mice. The rectangular dashed box marks the region of interest, which is shown at higher magnification in the inset. An arrowhead points to the vessel which is rendered in 3D. NOTCH3 ECD aggregates on vessels are depicted by arrows. (**B**) Light sheet fluorescence microscopy (LSFM) imaging of arteries (stained with α-SMA) in the hippocampus of 9-month-old mice, as indicated after iDISCO+ clearing, imaged at ×6.4 magnification (*top*). The rectangular dashed boxes mark the regions of interest, which are shown at higher magnification in the *inset*. Vessels that have lost SMA staining are indicated with arrows. *Right*: α-SMA masked surface rendering in the hippocampus region. (**C**) Quantification ratio of α-SMA loss with Imaris 10.2.0. In the WT-TgN3 group, the SMA surface is generated and displayed in green, while in the R182C-TgN3 group, the SMA surface is shown in transparent white. The green SMA surface in R182C-TgN3 was derived by using the same intensity threshold as WT-TgN3 mice. The SMA loss ratio was quantified as the volume of the green SMA surface divided by the volume of the transparent white SMA surface. Bar graph shows mean ± standard error of the mean. An unpaired two-tailed Student’s *t*-test was used for statistical analysis. *n* = 5 per genotype, ****P < 0.0001. CADASIL = cerebral autosomal dominant arteriopathy with subcortical infarcts and leukoencephalopathy; iDISCO+ = immunolabeling-enabled 3D imaging of solvent-cleared organs; VSMC = vascular smooth muscle cell; WT = wild-type.

Adequate blood flow is crucial for maintaining the metabolic demands of neurons, especially during high-frequency activities like gamma oscillations. As CADASIL is a small vessel disease leading to reduced cerebral blood flow, we assessed whether the hippocampal vasculature was affected in a manner similar to changes previously observed in the cortex.^14,17^ We performed immunostaining using the 1E4 antibody to detect NOTCH3 ECD together with the vessel markers α-SMA (staining VSMCs) and CD31 [staining endothelial cells (EC)] on CADASIL mouse sections containing the hippocampal region. The tile imaging, which captured the entire hippocampal vasculature, revealed accumulation of NOTCH3 ECD in hippocampal capillaries and arterioles. Pronounced NOTCH3 ECD aggregates, along with loss of SMA staining in arterioles, were observed in the CADASIL mice (Fig. 6A). To obtain a more comprehensive view of the vascular network in the hippocampus, we deployed iDISCO+, a whole-mount immunolabelling protocol for SMA staining^31^ (Fig. 6B). The architecture of the hippocampal vascular network in the CADASIL mice showed a dramatic loss (60%–90% reduction) of SMA in the arch-like arteriole branches compared with control mice (Fig. 6C) (Supplementary Fig. 3 and Supplementary Video 1). As SMA is almost exclusively expressed in VSMCs, we interpret these data as extensive loss of VSMCs across the CADASIL mouse brain, including the hippocampus. This observation contrasts with one of our previous reports, which did not reveal a difference in SMA staining in 9-month-old mice.^32^ These different outcomes are likely attributable to the fact that we used the iDISCO+ 3D imaging technique, which is a much more sensitive method to record SMA content.

### Increased number and size of vessel-associated microglial cells in the hippocampus of CADASIL mice

Neuroinflammation is broadly defined as an inflammatory process in the brain or spinal cord which is not properly resolved.^33,34^ The microglial cells—the tissue-resident macrophages of the brain—play key roles in neuroinflammation and mitochondrial dysfunction and are thus an important cell type in neuroinflammation.^34,35^ While neuroinflammation has been extensively studied in neurodegenerative diseases, such as AD,^33^ the role of neuroinflammation in CADASIL remains relatively unexplored.

**Figure 7:**
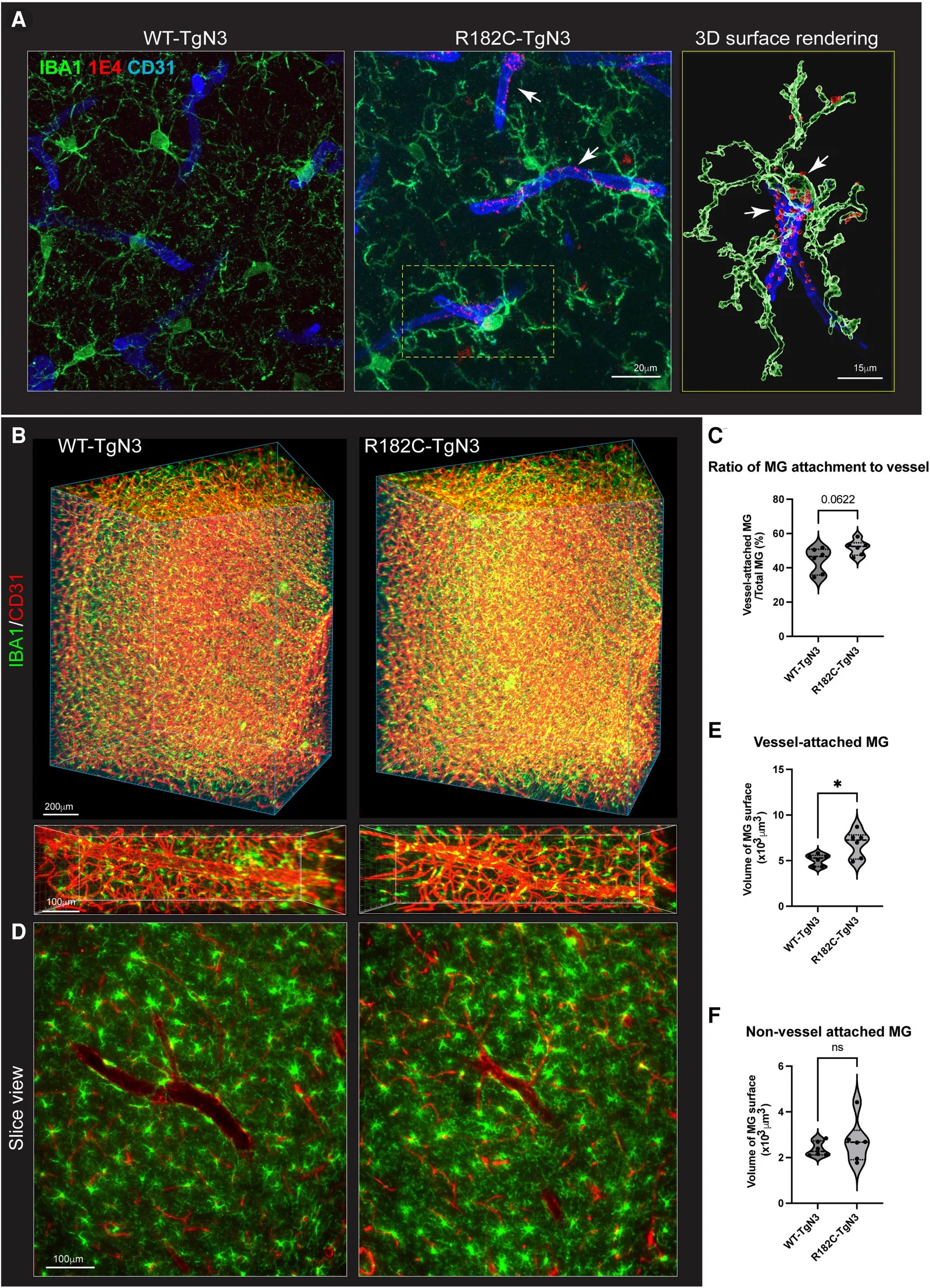
**Microglial alterations in CADASIL mice.** (**A**) Confocal images of the hippocampal region labelled with NOTCH3 extracellular domain (NOTCH3 ECD) (1E4 antibody, red), microglia (IBA1 antibody, green) and endothelial cells (CD31 antibody, blue) on sections from 9-month-old R182C-TgN3 and WT-TgN3 mice, as indicated. The rectangular dashed box marks the microglia, which are rendered in 3D and shown in the *inset*. The arrows denote the microglial phagocytosis of NOTCH3 ECD aggregates inside the soma **(B)** Light sheet fluorescence microscopy (LSFM) imaging of the hippocampus stained with CD31 and IBA1 after iDISCO+ clearing, imaged at ×8 magnification. *Top*: 3D views, and *bottom*: high magnification of segmented images. (**C**) Quantification of the number of vessel-attached microglia to total microglial cells. The bar graph was generated after surface rendering with Imaris. (**D**) Slice view of 3D imaging in the hippocampus region. (**E**) Quantification of volume of vessel-attached microglia and (**F**) non-vessel-attached microglia after surface rendering using Imaris. Values are mean ± standard error of the mean; unpaired two-tailed Student *t*-test was used for statistical analysis, *P < 0.05, **P < 0.01, ns = non-significant. CADASIL = cerebral autosomal dominant arteriopathy with subcortical infarcts and leukoencephalopathy; iDISCO+ = immunolabeling-enabled 3D imaging of solvent-cleared organs; MG = microglia; WT = wild-type.

To explore the microglial cells in the CADASIL mice, we investigated the spatial relationship between the NOTCH3 ECD deposits and microglial cells in the CADASIL hippocampus. Immunohistochemistry analysis of hippocampal sections from the CADASIL mice showed 1E4-positive NOTCH3 ECD aggregates within the soma of the microglial cells, visualized by staining with the microglial marker IBA1 (Fig. 7A). To gain further insight into how NOTCH3 ECD accumulation in the vessels affects microglial cells, we performed iDISCO + analysis on whole mouse brains stained with IBA1 and the endothelial cell marker CD31 (Fig. 7B). In the hippocampal region, the number of IBA1+ cells attached to the CD31+ endothelial cells was significantly increased in the CADASIL mice compared with control mice (Fig. 7C). Furthermore, a hippocampal slice view from the 3D iDISCO image (Fig. 7D) showed a significant increase in the size of the IBA1+ cell bodies attached to vessels (Fig. 7E), whereas the cell body size of the non-vessel attached microglial cells was not changed compared with control mice (Fig. 7F) (Supplementary Video 2). To analyse the interaction between microglial cells and the vasculature in the hippocampus in further detail, we assessed the microglial cells in the hippocampus of CADASIL mice by transmission electron microscopy (TEM). We observed that microglial cells were in close vicinity to the vessel wall and that they are capable of phagocytosing large aggregates, possibly including NOTCH3 ECD deposits (Supplementary Fig. 4). Together, these data indicate that the number and size of microglial cells is increased in the CADASIL mice and contain NOTCH3 aggregates.

### Microglia subcluster enriched for mitochondrial and inflammatory genes in CADASIL mice

**Figure 8:**
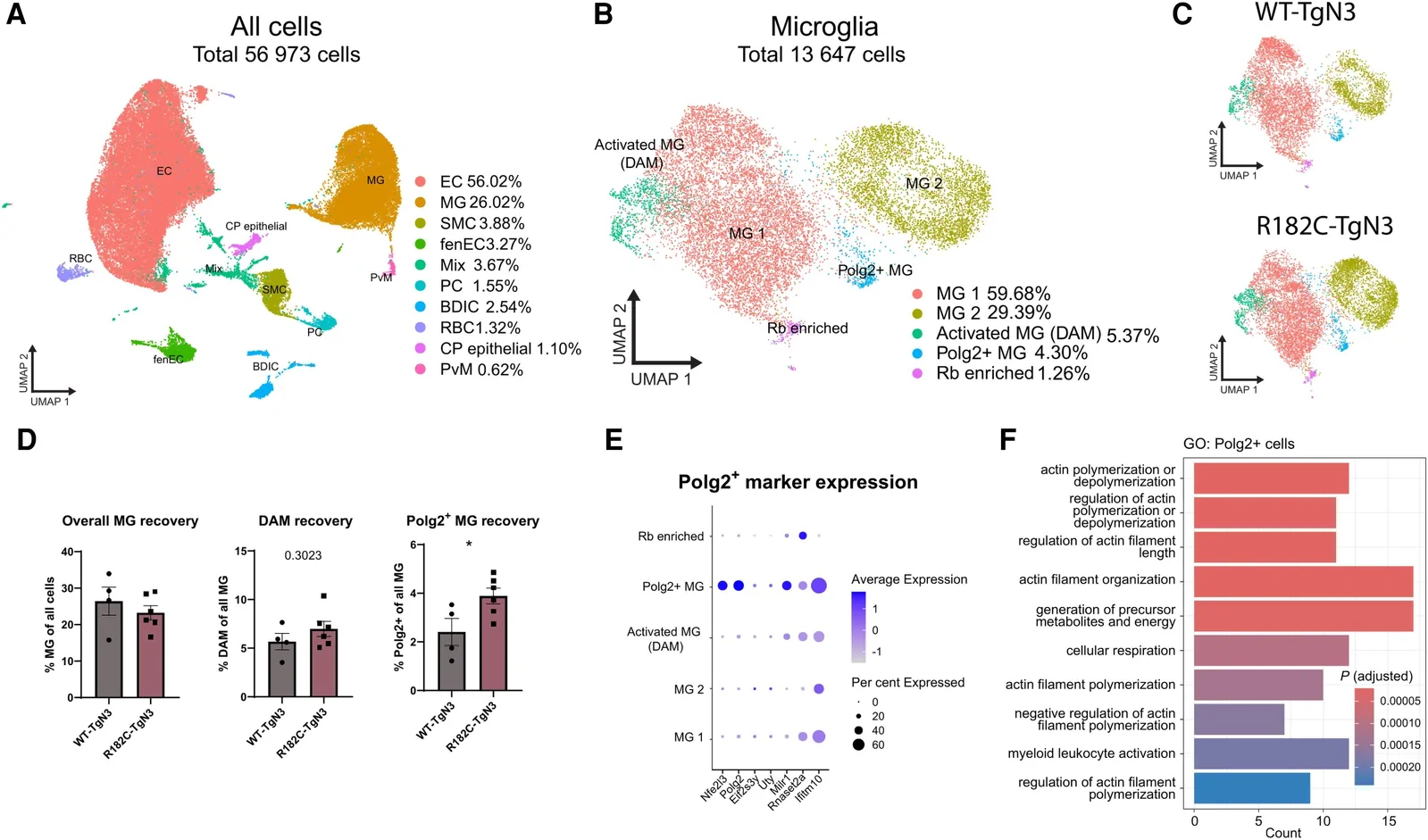
**Single-cell sequencing of isolated cerebral microvessels from R182C-TgN3 and WT-TgN3 mice.** (**A**) Uniform manifold association plot (UMAP) of all cells isolated from all experimental conditions. Labels depict the annotation of superclusters based on the marker genes in each supercluster. Cell-specific proportions across all samples are given as the percentage of all cells in the legend. **(B**) UMAP of microglial cells after isolation from the whole dataset. (**C**) UMAP of microglial cells split in experimental conditions. (**D**) Sampling variation of microglia (*left*), DAMs (*middle*) and Polg2+ microglia. Each dot represents a sample percentage of all cells or all microglia. Error bars represent mean ± standard error of the mean. Students *t*-test. (**E**) Dot plot showing the expression of the most specifically expressed genes in the Polg2 cluster across all microglial cells. (**F**) Gene ontology (GO) terms enriched of the genes in the *Polg2* subcluster of R182C-TgN3. BDIC = blood-derived immune cells; CP epithelial = choroid plexus epithelial cells; DAM = disease-associated microglia; EC = endothelial cells; fenEC = fenestrated endothelial cells; MG = microglia; PC = pericytes; PvM = perivascular microglial; RBC = red blood cells; Rb enriched = ribosome-enriched microglias; SMC = smooth muscle cells; WT = wild-type.

To shed further light on the vascular-associated microglia and their crosstalk with the endothelial and mural cells we conducted single-cell RNA sequencing (scRNAseq) on vascular and vasculature-associated cells from the CADASIL mice using a recently established protocol for the isolation of cerebrovascular cells.^20^ We isolated a total of 56 973 cells from 6–9-month-old CADASIL mice and control mice after initial QC. The cells distributed into 10 annotated subclusters [31 918 endothelial cells, 3094 mural cells and 14 826 vascular-associated microglia (MG)] (Fig. 8A and Supplementary Fig. 5A–C). Upon isolation and further quality control of the MG supercluster 13 647 microglial cells were analysed (Fig. 8B). Annotation using subcluster-specific markers resulted in the identification of five subclusters, representing two separate microglial clusters (MG 1 and 2); a specialized subtype called disease-associated microglia (DAMs) characterized by the expression of *Clec7a* and *Apoe*; a cluster expressing higher levels of *Polg2* and *Nfe2l2*; and a ribosome-enriched cluster. All identified subclusters were represented in both CADASIL and control mice (Fig. 8C) and there was no difference in overall sampling of microglia between CADASIL and control mice (Fig. 8C and D), as well as no difference in the recovery of MG 1 and MG 2 or DAMs (Fig. 8D and Supplementary Fig. 5D). There was, however, a significant increase in the number of *Polg2*-positive microglia in CADASIL compared with control mice (Fig. 8D). *Polg2* is a marker for mitochondrial respiration^36^ and analysis of the marker genes of this subcluster showed that *Polg2, Nfe2l2* (involved in oxidative stress and inflammation), and *MIlr2* (involved in immune response), were specific to this microglial subcluster (Fig. 8E). Gene ontology (GO) analysis of the *Polg2* cluster marker genes indeed revealed changes in the cellular respiration pathway (Fig. 8F). Furthermore, the GO analysis indicated changes in terms of regulation of actin filament polymerization and organization, indicating that cell morphology and size may be affected. In sum, the data provide support for mitochondrial impairment and morphological changes in the microglial cells in the CADASIL mice.

## Discussion

CADASIL is the most common monogenic form of cerebral small vessel disease, and while the vessel wall pathology, clinical and neuroimaging aspects of the disease have been extensively investigated, little is known about the specific role of the hippocampus or the detailed metabolic and inflammatory effects on neurons and vascular cells. To address this, we have used a transgenic mouse model together with post-mortem CADASIL patient brain samples and cell lines to explore possible neuronal dysfunction in CADASIL. Our data reveal that the CADASIL mouse hippocampus exhibited a significant decrease in gamma oscillation power, combined with an increase in frequency variance. As gamma oscillation alterations are also observed in mouse models for Alzheimer’s disease,^37^ we speculate that the gamma oscillation deviations observed in the CADASIL mice are likely also associated with neuronal dysfunction in the hippocampus of the CADASIL mice, although any potential causality will need to be addressed in future studies. In support of this notion, expression of the neuronal markers NFH and UCHL1 was reduced in the CADASIL mice as well as in post-mortem brain tissue from CADASIL patients carrying different *NOTCH3* variants. Furthermore, nerve fibres in both the CADASIL mice and post-mortem CADASIL patient tissue were disorganized and neurons exhibited a runted morphology, further indicating extensive neuronal aberrations in CADASIL and corroborating recent findings of reduced numbers of neurons in the hippocampus of CADASIL patients.^38^ It is, however, of note that despite the neuronal effects in the CADASIL mice, a previous study did not reveal any cognitive or motor changes in the same CADASIL mouse model.^32^We hypothesize that this may be because, even at 20 months of age, the mice are still in a pre-clinical disease stage, as they have not yet developed brain parenchymal pathology.^14^

It seems reasonable to assume that the CADASIL disease process starts in the brain vasculature, as the NOTCH3 receptor is almost exclusively expressed in VSMCs and pericytes in the brain. Therefore, further insights into the vascular dysfunction in the CADASIL mice are warranted in order to understand how vascular problems may lead to the observed neuronal consequences. NOTCH3 ECD aggregates were observed around hippocampal capillaries and arterioles, accompanied by vascular dysfunction in the CADASIL mice. Notably, the vascular network was significantly sparser in the hippocampus of CADASIL mice as compared with controls, and the number of VSMCs, as judged by loss of SMA staining in iDISCO+ immunolabelling, was similarly reduced by 60%–90% in hippocampal arterioles. A loss of VSMCs was corroborated by reduced SMA levels in hippocampal lysates from CADASIL mice, as judged by western blot analysis, combined with increased expression of MCT1 [a protein that primary facilitates lactate import into cells (data not shown)].^39^ The increase in MCT1 levels may indicate a compensatory metabolic response to sustain neuronal energy demands in the face of the dysfunction of mitochondrial complexes, as enhanced monocarboxylate transport can support glycolysis-dependent ATP production and facilitate lactate shuttling between vascular/glial cells and neurons. We furthermore did not observe blood–brain barrier leakage, determined by staining for plasma fibrinogen (data not shown) or loss of pericyte coverage by staining for pericyte and endothelial cell markers (data not shown) in the CADASIL mouse model. These data are in line with previous observations in both CADASIL mice and CADASIL patients.^14,32,40^

There are several mechanisms by which these extensive vascular aberrations may subsequently lead to the observed neuronal problems. One obvious mechanism is that the pathological vasculature create a hypoxic situation, which in turn affects the neurons in the CADASIL mice, in particular as there is a long distance between blood vessels and the tightly packed pyramidal neurons in the hippocampus, possibly making the neurons particularly vulnerable to a hypoxic assault. Several studies using brain MRI in CADASIL patients show abnormalities in the function of the small vessels as a consequence of stiffness of the arterioles and impaired regional reactivity in a blood-oxygen-level dependent manner.^41-43^

Impaired mitochondrial function in the vasculature could be a possible link between vascular ischaemia and neuronal dysfunction. In support of this hypothesis, we observed that all five respiratory complexes were affected in more than one sample source (complex I in isolated vessels and human VSMC, complex II in all three sample sources, complex III in hippocampal lysates and isolated vessels, complex V in isolated vessels and human VSMC). It should be noted that the levels of respiratory complex II and III were reduced in isolated vascular fragments and hippocampal lysates from the CADASIL mice, suggesting that vascular defects may contribute to the mitochondrial impairment. Similarly, in the *in vitro* cultured VSMCs carrying the R133C *NOTCH3* variant, respiratory complex I, II and V levels were reduced, accompanied by a reduction in basal respiration and ATP production. These data corroborate previous reports suggesting that human CADASIL VSMCs exhibit an abnormal mitochondrial morphology.^44^ It is also noteworthy that complex II is significantly reduced in all three types of samples and deficiency in complex II is also linked to primary mitochondrial disorders, in which patients’ brains typically exhibit white matter and capillary abnormalities, selective neuronal loss, and lesions resembling those seen in CADASIL mice and indistinguishable from small partial infarctions.^45^ Therefore, mitochondrial impairment could contribute to the broader neurovascular and neurodegenerative changes observed in CADASIL. A third potential link between an ailing vasculature and dysfunctional neurons is neuroinflammation, and our data indeed provide evidence for a low-grade neuroinflammatory process in the CADASIL brains. We found that the human CADASIL VSMCs expressed high levels of pro-inflammatory markers, suggesting that inflammation plays an important role in CADASIL aetiology, which is in line with previous work.^16,46^ Furthermore, there was an increase in vessel-associated microglial cells, accompanied by an increase in microglial cell body size in the CADASIL mouse model, and the accumulation of microglial cells near the cerebrovasculature may be a result of them being attracted to the vessels by the pro-inflammatory substances secreted by the VSMCs. Moreover, microglia were found to be engaged in phagocytosis of NOTCH3 ECD deposits, which may further trigger a neuroinflammatory response. Cytokines, such as Tumor necrosis factor-alpha (TNF-α) and Interleukin-6 (IL-6), were also elevated in serum from the CADASIL mouse model (data not shown), indicating that the neuroinflammation is not only confined to the brain but may be more systemic, which is in line with a previous report showing that there is an increase of inflammation markers in the plasma from CADASIL patients using an alternative assay (the Olink platform, measuring 552 proteins across six panels).^47^ The discovery of a *Polg2*+ microglial subcluster further underscores that microglia are activated in CADASIL. However, to what extent the microglial activation plays a role in neuronal dysfunction in CADASIL remains to be established.

In conclusion, we shed new light on the neuronal aspects of CADASIL and provide evidence for an altered gamma oscillation pattern and neuronal aberrations in CADASIL mice and human post-mortem tissue and cell lines. We also provide new insights into the vascular dysfunction in CADASIL. Based on this combined analysis of neurons and the vasculature, our data point to an interplay between hypoxia, mitochondrial impairment and neuroinflammation as potential factors that may convey signals from an ailing vasculature to the nervous system, leading to the observed neuronal aberrations. These findings are important for a better understanding of the pathophysiology of CADASIL and may inform on future therapeutic avenues to retain vascular function, by for instance treating patients with low doses of anti-inflammatory drugs that may slow down disease progression.

## Supplementary Material

awag033_Supplementary_Data

## Data Availability

The single-cell RNA sequencing raw data are publicly available at the Gene Expression Omnibus on accession number GSE300114.
