# Supplementary material for: Impairment of hippocampal gamma oscillations, mitochondria and neurovascular function in CADASIL
Source: Brain. 2026 Feb 2;149(9):3031–45. doi: 10.1093/brain/awag033 (PMC13548857; doi:10.1093/brain/awag033)

**Supplementary Material.** Full-length Western blots from Figure 2B, H and Figure 5A, C, E. Supplied in a separate file.

**Supplementary Tables 1-3 supplied in a separate file.**

**Supplementary Video 1.** 3D movie of iDISCO+ cleared hippocampus of 9-month-old WT-TgN3 and R182C-TgN3 mice stained with SMA using LSFM imaging and 3D rending SMA surface generated with Imaris.

**Supplementary Video 2. Microglia alterations in the CADASIL mice.** 3D movie of microglia and vessel attachment in the hippocampus region at a higher magnification from 3D view and sectional view.

**Supplementary figure 1. Loss of neuronal fibers and altered neuronal morphology in human CADASIL brains.** Immunohistochemical analysis of UCHL1 **(A)** and **(B)** NFH staining in histological prefrontal cortical sections (10X left panels), 20X right panels from control and additional CADASIL patients than in Figure 3 with the p.R133C, p.R141C and p.C455R *NOTCH3* variants, sections were counterstained by hematoxylin.

**Supplementary figure 1. Loss of neuronal fibers and altered neuronal morphology in human CADASIL brains.**

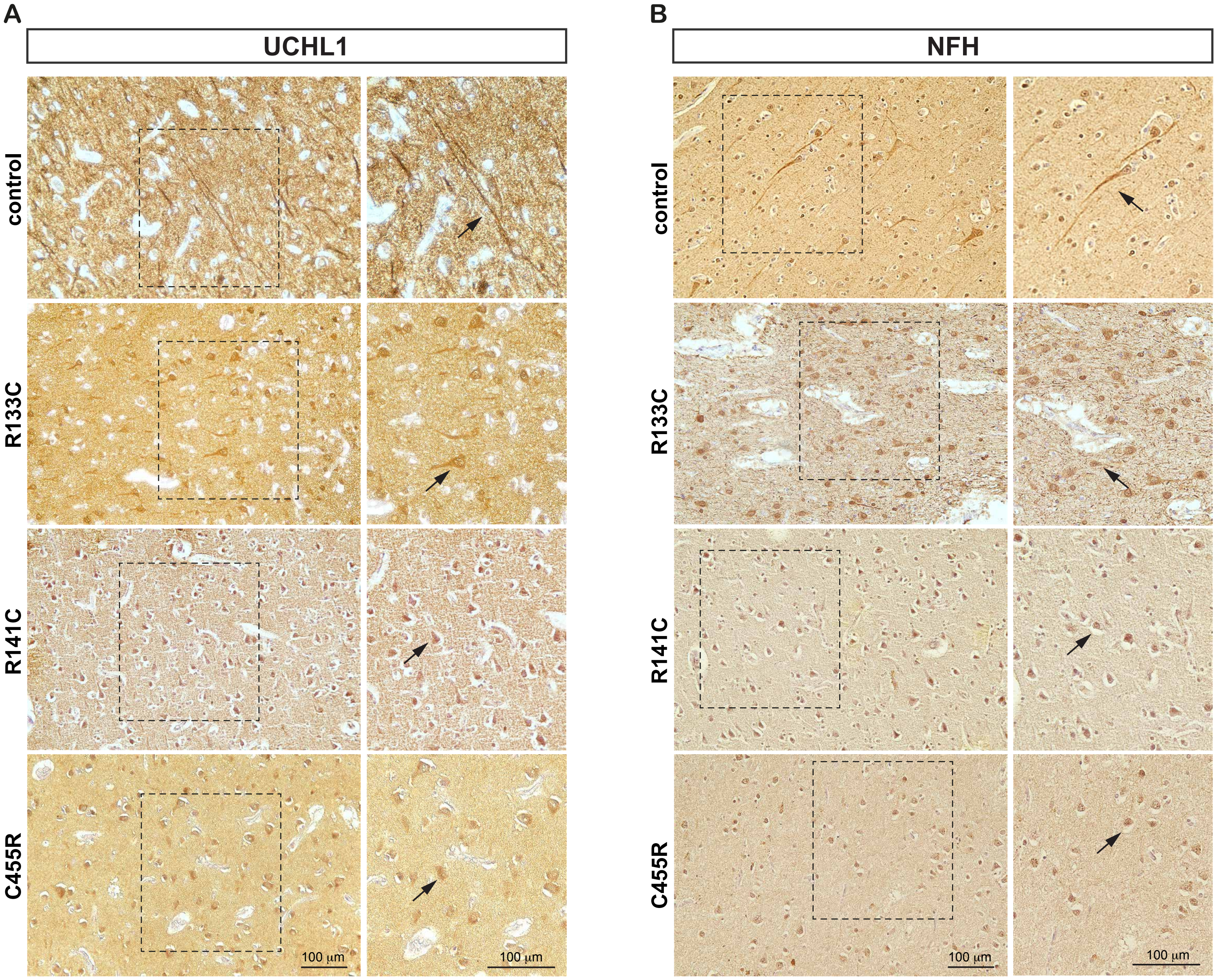

**Supplementary figure 2. Cellular evaluation of excitatory pyramidal cells and inhibitory fast-spiking interneurons in hippocampus CA3**. (**A-D)** Excitatory pyramidal cells. (**A**) Membrane potential (*P*= 0.3557). **(B)** Excitatory postsynaptic currents charge transfer. **(C)** Vector length. **(D)** Firing rate. **(E-H).** Inhibitory fast-spiking interneurons. **(E)** Membrane potential *P*= 0.3095). **(F)** Excitatory postsynaptic currents charge transfer. **(G)** Vector length. **(H)** Firing rate. Data were presented as means ± S.E.M in bar graphs. E, F, G and K were analysed by using unpaired two-tailed Student's *t*-test. H, I, J and L were analysed using Mann Whitney test. The number of animals used in the experiment were presented as dots in the bar graphs.

**Supplementary figure 2. Cellular evaluation of excitatory pyramidal cells and inhibitory fast-spiking interneurons in hippocampus CA3.**

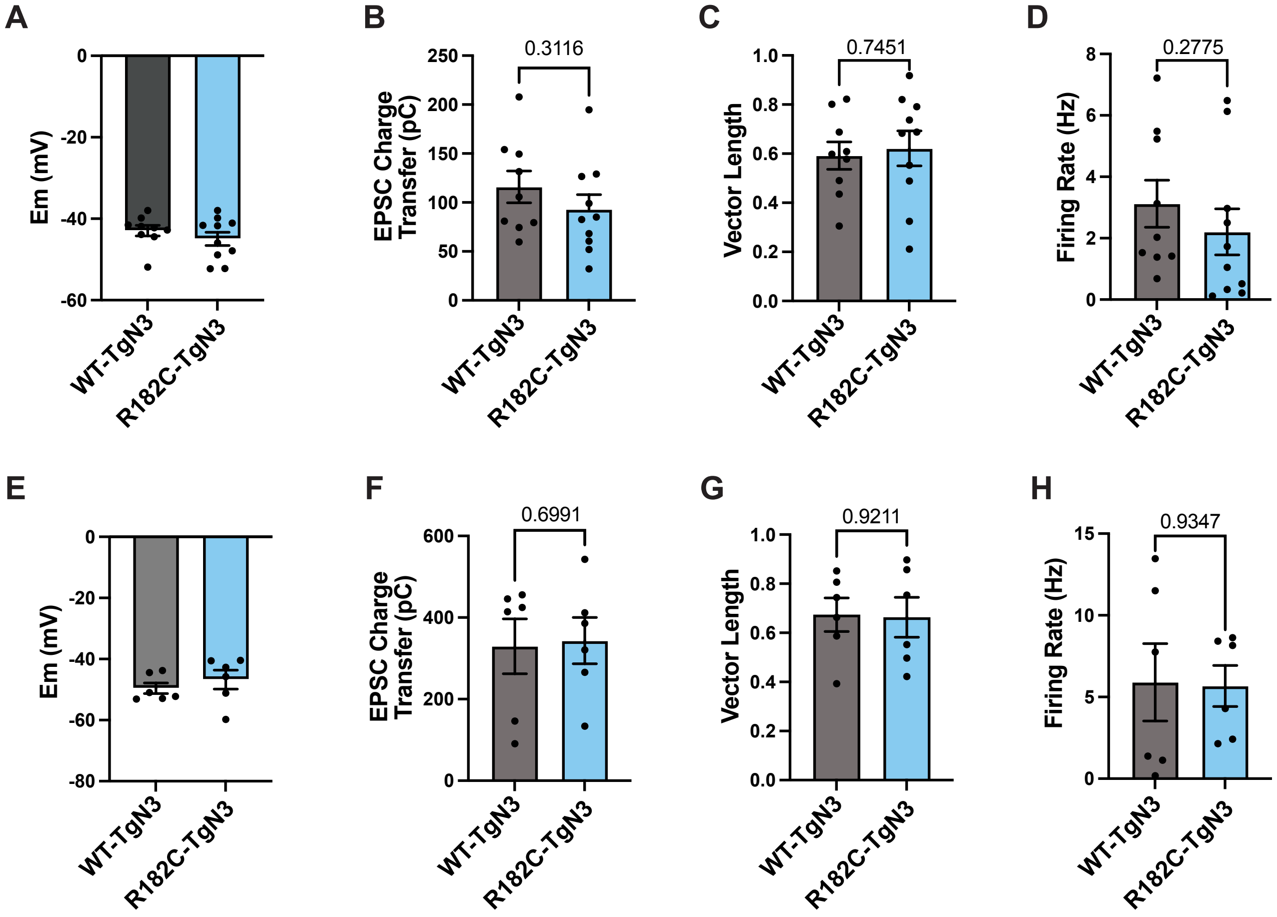

**Supplementary figure 3. Hippocampal artery pathology in the CADASIL mice**. LSFM imaging at 3.2X magnification of the hippocampus stained with SMA followed by iDISCO+ clearing of 9-month-old R182C-TgN3 mice.

**Supplementary figure 3. Hippocampal artery pathology in the CADASIL mice.**

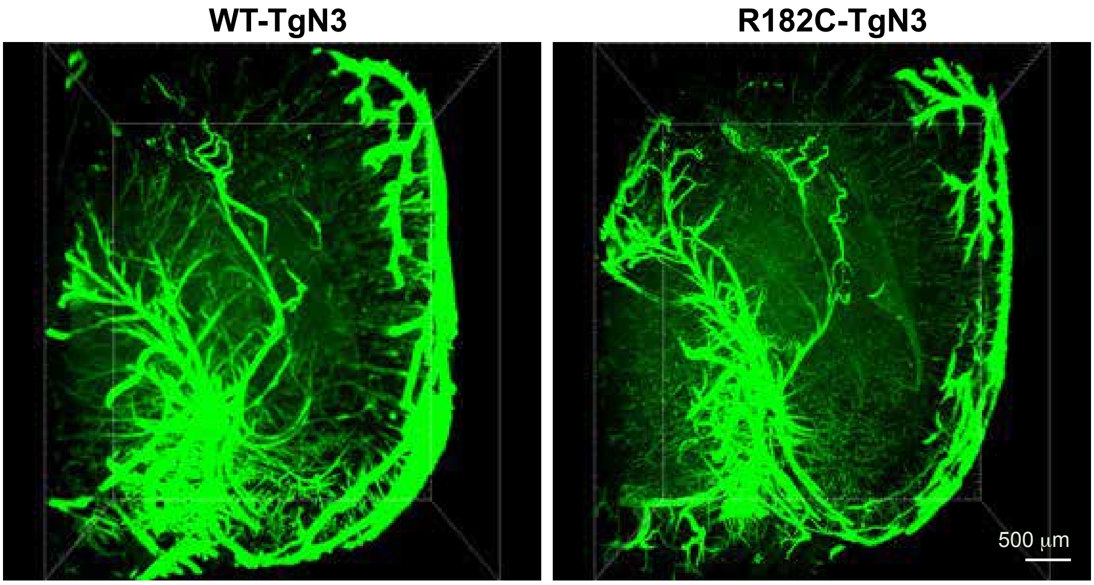

**Supplementary figure 4. Microglia alterations in the CADASIL mice.** Electron microscopy image of hippocampal region from 9-month-old WT-TgN3 and R182C-TgN3 mice where microglial cells (m) can be found in close vicinity to the vessel wall (arrowhead) and containing granular material, possible NOTCH3 ECD deposits (*).

**Supplementary figure 4. Microglia alterations in the CADASIL mice.**

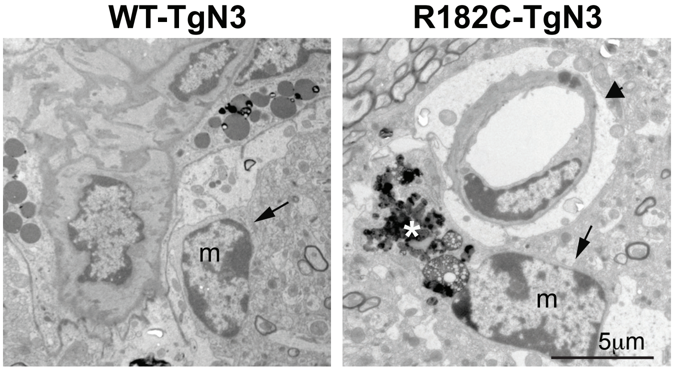

**Supplementary figure 5. Single cell sequencing of isolated cerebral microvessels from R182C-TgN3 and WT-TgN3 mice. (A)** Uniform manifold association plot (UMAP) of all cells isolated from all experimental conditions. Labels depict the annotation of superclusters based on the marker genes in each supercluster split in the two experimental conditions. **(B)** Panel showing overall single cell recovery in number of cells per sample (upper left) and relative recovery (% of all sampled cells) of endothelial cells (ECs), smooth muscle cells (SMC), and pericytes (PC) from the two experimental conditions. Data is shown as individual values, mean and SEM. Tested by Students T-test. **(C)** Dotplot showing relevant marker genes for annotation of the superclusters in Figure 8A. **(D)** Recovery of the two microglia (MG) subclusters MG1 and MG2. Data shown as in **(B)**.

## Supplementary figure 5. Single cell sequencing of isolated cerebral microvessels from R182C-TgN3 and WT-TgN3 mice.

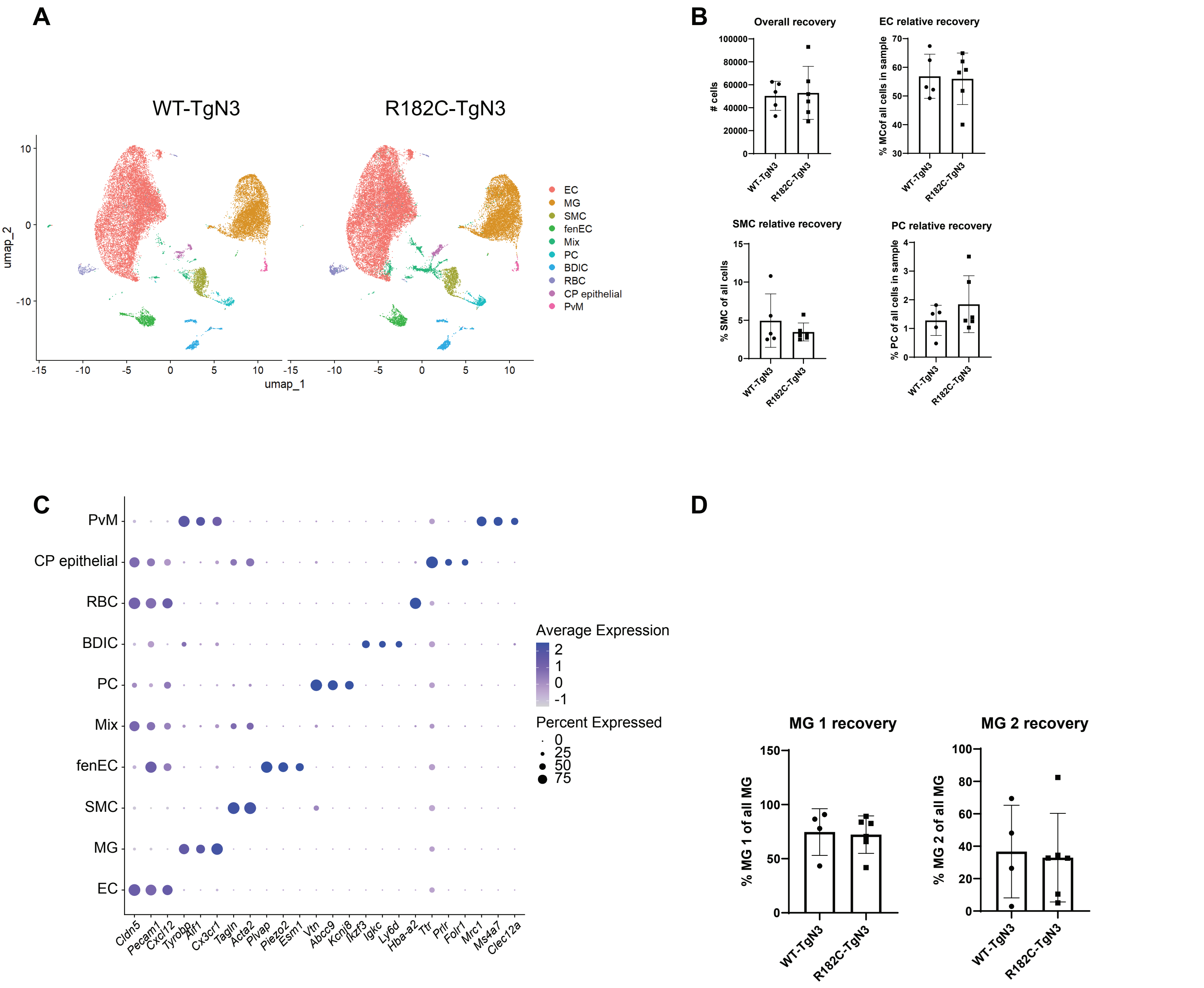

Supplement: Supplementary file 1 [file awag033_supplementary_data.zip › brain-2025-01754-File010.pdf]
