# Supplementary material for: Impairment of hippocampal gamma oscillations, mitochondria and neurovascular function in CADASIL
Source: Brain. 2026 Feb 2;149(9):3031–45. doi: 10.1093/brain/awag033 (PMC13548857; doi:10.1093/brain/awag033)
Supplement: Supplementary file 1 [file awag033_supplementary_data.zip › brain-2025-01754-File013.pdf]

Supplementary Table 1. Antibodies and reagents

Antibodies	Company	Catalog Number
Anti-NeuN antibody	Abcam	AB177487
CD68/SR-D1 Antibody (FA-11)	BIO-RAD	NBP2-33337
Postsynaptic density protein 95 antibody	Cell Signaling Technology	3450
Synaptophysin antibody	Cell Signaling Technology	4329
Neurofilament heavy chain antibody	DSHB	RT97-C
Anti IBA1	FUJIFIKM Wako	4987481428584
Donkey anti-Goat IgG (H+L) Highly Cross-Adsorbed Secondary Antibody, Alexa Fluor™ Plus 488	Invitrogen™	A32814
IRDye® 680RD Donkey anti-Mouse IgG Secondary Antibody	LICORbio™	926-68072
IRDye® 800CW Donkey anti-Mouse IgG Secondary Antibody	LICORbio™	926-32212
IRDye® 800CW Donkey anti-Rabbit IgG Secondary Antibody	LICORbio™	926-32213
IRDye® 800CW Goat anti-Mouse IgG Secondary Antibody	LICORbio™	926-32210
Alexa Fluor® 647-conjugated AffiniPure™ F(ab')2 Fragment Donkey Anti-Goat IgG (H+L)	Nordic Biosite	705-606-147
Cy3-conjugated AffiniPure™ F(ab')2 Fragment Donkey Anti-Rabbit IgG (H+L)	Nordic Biosite	711-166-152
Alexa Fluor® 647-conjugated AffiniPure™ F(ab')2 Fragment Donkey Anti-Rat IgG (H+L)	Nordic Biosite	712-606-150
Mouse/Rat CD31/PECAM-1 Antibody	R&D Systems	AF3628BHV HB
Anti-NOTCH 3/N3ECD Antibody, clone 1E4	Sigma-Aldrich	MABC594
Actin polyclonal antibody	Thermo Scientific™	PA5-78716
GAPDH polyclonal antibody	Thermo Scientific™	A1-988
Lysosomal-associated membrane protein-1	Thermo Scientific™	14-1079-80
OxPhos rodent WB antibody Cocktail	Thermo Scientific™	45-8099
UCHL1 monoclonal antibody (BH7)	Thermo Scientific™	480012
β-actin monoclonal antibody	Thermo Scientific™	MA1-140
Horse Anti-Mouse IgG Antibody (H+L), DyLight™ 594	Vector Laboratories	DI-2594-1.5
Horse Anti-Rabbit IgG Antibody (H+L), DyLight™ 594	Vector Laboratories	DI-1094-1.5

Reagents	Company	Catalog Number
4–15% Mini-PROTEAN® TGX™ Precast Protein Gels	BIO-RAD	4561085
Trans-Blot Turbo RTA Mini 0.2um PVDF Transfer Kit	BIO-RAD	1704272
XT Sample Buffer (4X)	BIO-RAD	161-0791
Diva Decloaker (10X)	Biocare Medical	DV2004MX
MACH 3 Mouse HRP-Polymer Detection	Biocare Medical	M3M530L
MACH 3 Rabbit HRP Polymer Detection	Biocare Medical	M3R531L
Background Sniper	Biocare Medical	BS966L
Betazoid DAB Buffer	Biocare Medical	DS900H
DMEM	Gibco™	D5030
L-Glutamine (200 mM)	Gibco™	A2916801
Smooth muscle growth Supplement (SMGS)	Gibco™	S00725
BenchStable™ DMEM/F12	Gibco™	A4192002
Mouse RANTES Uncoated ELISA Kit	Invitrogen™	88-56009-88
Sodium Dodecyl Sulfate (SDS)	Invitrogen™	15525017
RNeasy mini kit Mouse RANTES Uncoated ELISA Kit	QIAGENInvitrogen™	74104 88-56009-88
Carbonyl cyanide-p-(trifluoromethoxy) phenylhydrazone (FCCP)	Sigma-Aldrich	C2920
Dibenzyl ether (DBE)	Sigma-Aldrich	33630
Dichloromethane (DCM)	Sigma-Aldrich	5.89581
2-deoxy-D-glucose	Sigma-Aldrich	D8375
Hydrogen peroxide solution	Sigma-Aldrich	102704116
Oligomycin A	Sigma-Aldrich	75351
Paraformaldehyde (PFA)	Sigma-Aldrich	158127
Rotenone	Sigma-Aldrich	R8875
Antimycin A	Sigma-Aldrich	A8674
Bovine Serum Albumin lyophilized powder (BSA)	Sigma-Aldrich	A2153
Dithiothreitol (DTT)	Thermo Scientific™	R0861
Glycerol	Thermo Scientific™	332031000
Maxima First Strand cDNA Synthesis Kit for RT-qPCR	Thermo Scientific™	K1642
Phosphatase and protease inhibitor	Thermo Scientific™	A32953
Pierce BCA Protein Assay Kit	Thermo Scientific™	23225
SuperBlock™ Blocking Buffer	Thermo Scientific™	37515
SYBR Green master mix	Thermo Scientific™	A46012
Bromophenol Blue, ACS	Thermo Scientific™	032641.03

Supplementary Table II. Primers for qPCR analysis

**Gene**	**Primer sequence**
CCL2.Forward	GCTCAGCCAGATGCAATCAA
CCL2.Reverse	TTCTTTGGGACACTTGCTGC
CCL5.Forward	CCAGCAGTCGTCTTTGTCAC
CCL5.Reverse	CTCTGGGTTGGCACACACTT
ICAM1.Forward	ATGCCCAGACATCTGTGTCC
ICAM1.Reverse	GGGGTCTCTATGCCCAACAA
IL8.Forward	GGTGCAGTTTTGCCAAGGAG
IL8.Reverse	TTCCTTGGGGTCCAGACAGA
ANGPTL4.Forward	GTCCACCGACCTCCCGTTA
ANGPTL4.Revserse	CCTCATGGTCTAGGTGCTTGT

Supplementary Table III. Clinical data of human CADASIL patients and controls.

For figure 3.

Case no	Mut	Sex	Age	Disease (years)	Disease stage and MRI	Cognitive profile
OxCad/01-K	p.R133C	F	53	6	Subcortical infarcts including basal ganglia and white matter; Gliosis	Mild Vascular Cognitive Impairment
NA56/06	p.R133C	F	68	17	Subcortical infarcts including basal ganglia and white matter; Arteriosclerotic thickening	Severe Vascular Cognitive Impairment
NA271/99-AG	p.R169C	M	61	10	Subcortical infarcts including basal ganglia and white matter; Intimal thickening in vessels	Severe Vascular Cognitive Impairment
NA87/05-AG	Control	F	68	N/A	No cerebrovascular or neurodegenerative disorders	No cognitive symptoms
C14-505-6	Control	M	47	N/A	No cerebrovascular or neurodegenerative disorders	No cognitive symptoms
S13-116-10	Control	F	18	N/A	No cerebrovascular or neurodegenerative disorders	No cognitive symptoms

For Supplementary fig 1.

Case no	Mut	Sex	Age	Disease (years)	Disease stage and MRI	Cognitive profile
R.H	p.R133C	M	63	10 (?)	Subcortical infarcts including basal ganglia and white matter. Periventricular white matter dilation	Moderate Vascular Cognitive Impairment
W/27	p.C455R	F	70	45	Subcortical infarcts including basal ganglia and white matter. Periventricular white matter dilation	Mild Vascular Cognitive Impairment
19870569	p.R141C	M	52	No data	Subcortical infarcts including BG and WM; Arteriosclerotic thickening	Severe Vascular Cognitive Impairment
C14-505-6	Control	M	47	N/A	No cerebrovascular or neurodegenerative disorders	No cognitive symptoms
C14-1032-8	Control	F	63	N/A	No cerebrovascular or neurodegenerative disorders	No cognitive symptoms
S13-116-10	Control	F	18	N/A	No cerebrovascular or neurodegenerative disorders	No cognitive symptoms
