# Supplementary material for: Impairment of hippocampal gamma oscillations, mitochondria and neurovascular function in CADASIL
Source: Brain. 2026 Feb 2;149(9):3031–45. doi: 10.1093/brain/awag033 (PMC13548857; doi:10.1093/brain/awag033)

Fig.2B

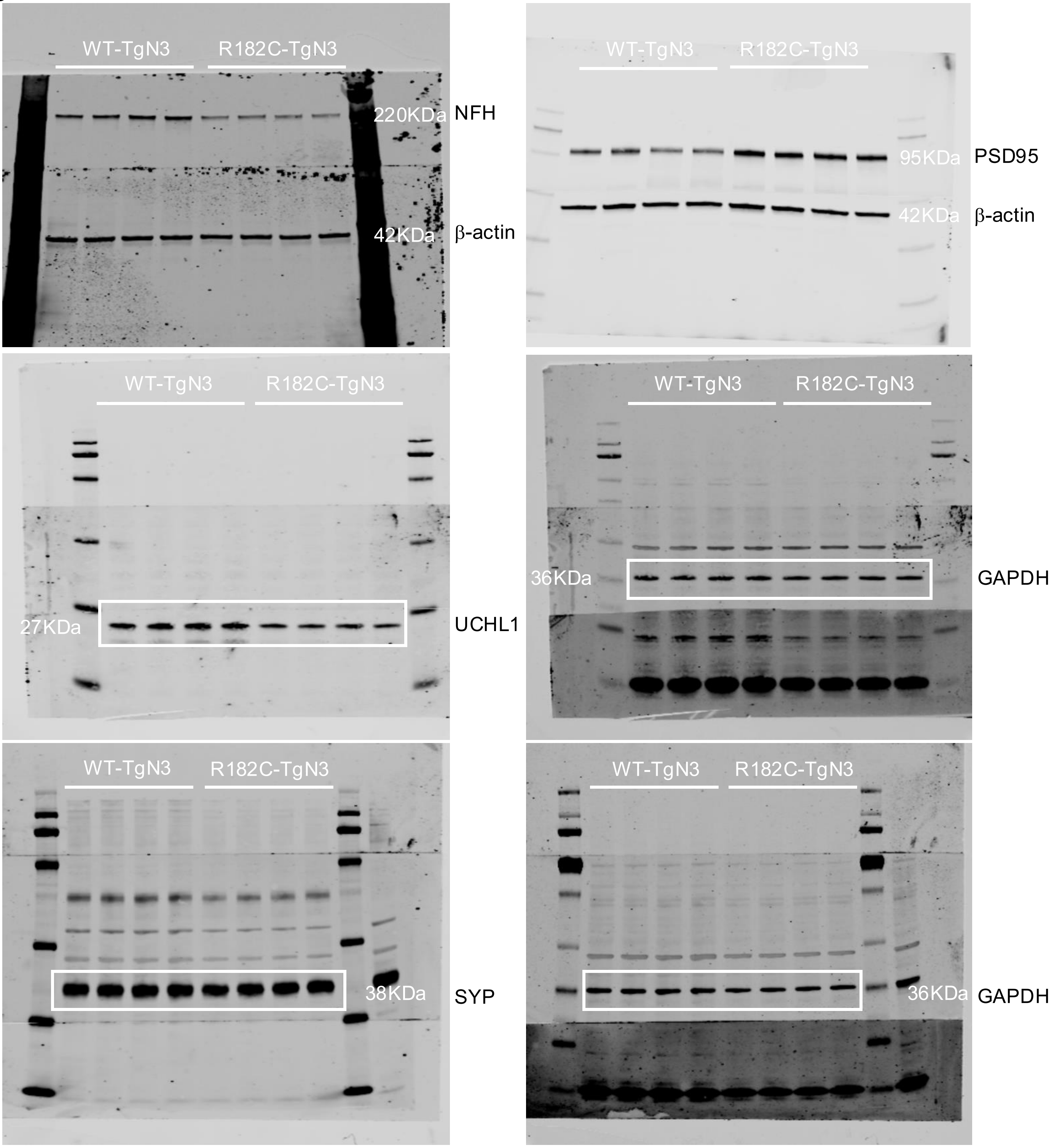
WT-TgN3
R182C-TgN3
220KDa NFH
42KDa β-actin
95KDa PSD95
42KDa β-actin
27KDa UCHL1
36KDa GAPDH
38KDa SYP
36KDa GAPDH

Fig.2H

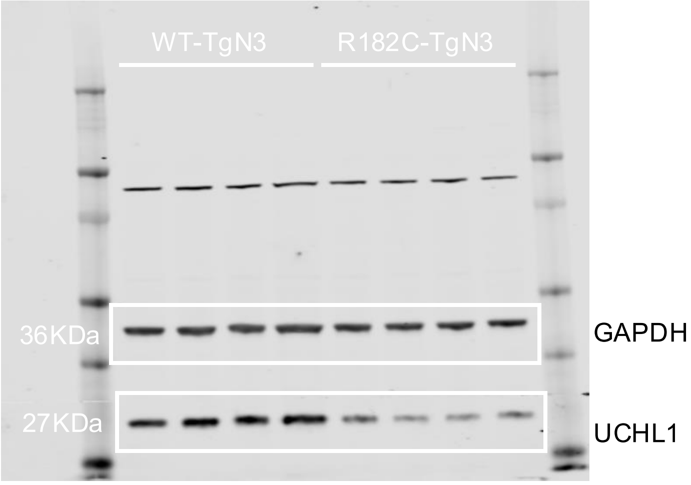
WT-TgN3
R182C-TgN3
36KDa GAPDH
27KDa UCHL1

Fig.5A

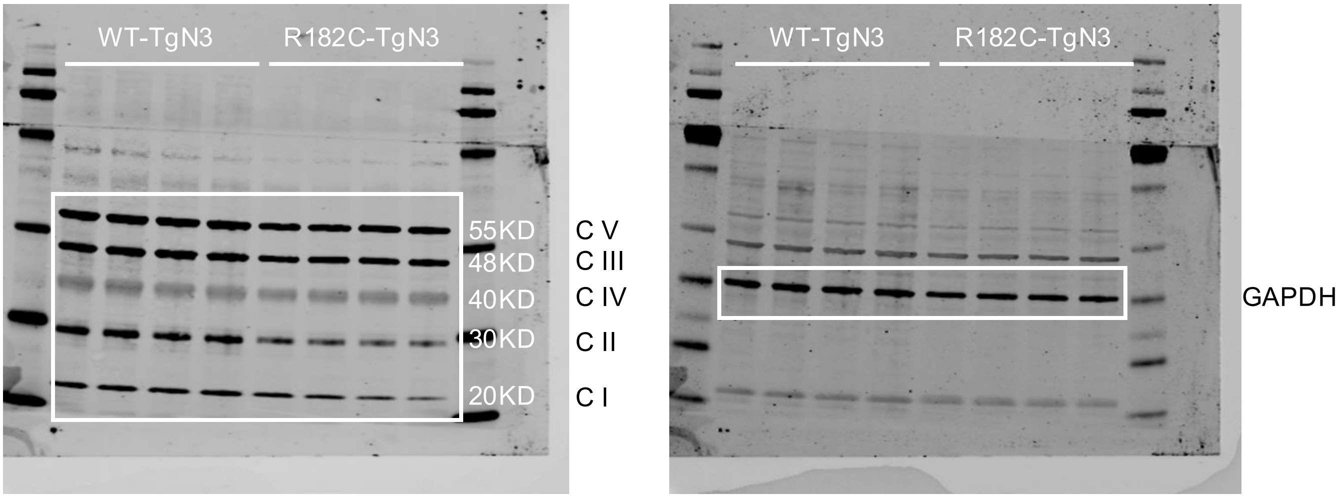

Fig.5C

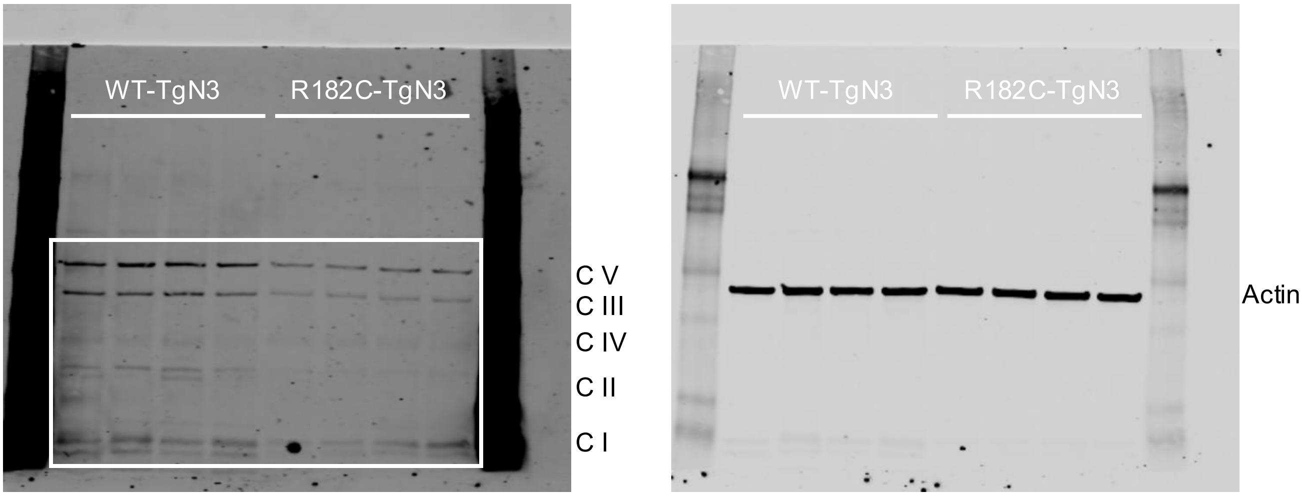

Fig.5E

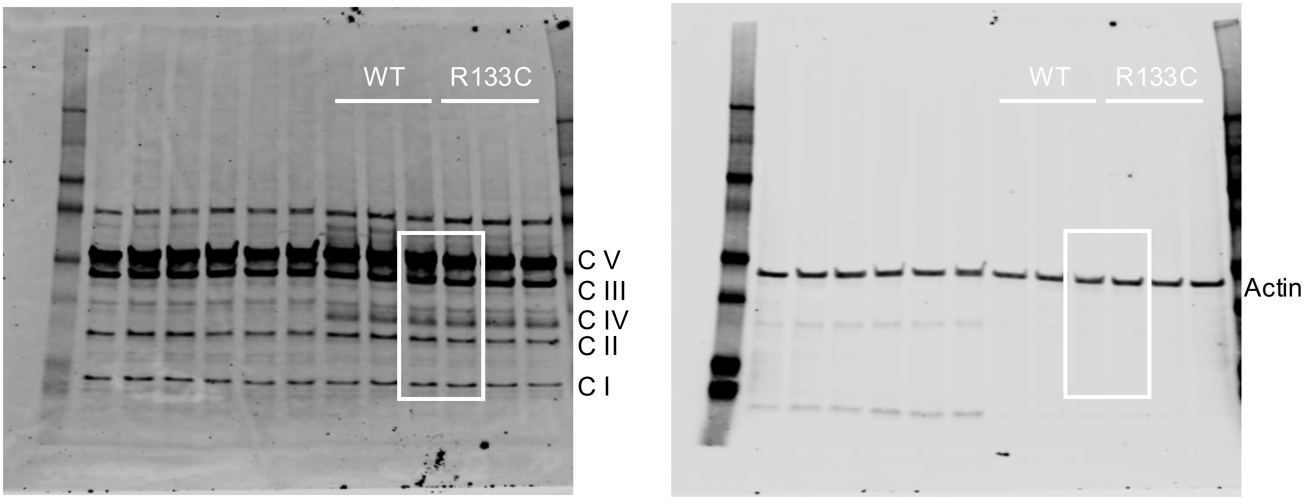

Supplement: Supplementary file 1 [file awag033_supplementary_data.zip › brain-2025-01754-File014.pdf]
